# Quantum Photothermal Self‐Monitoring Fiber Probes for In Vivo Photothermal Therapy

**DOI:** 10.1002/advs.75733

**Published:** 2026-05-20

**Authors:** Wanjun Li, Ruixiao Hu, Jie Mao, Jin Wo, Yunsong Zhang, Liang Chen, Ying Liu, Zhibo Li, Yongxin Lin, Minghui Du, Yaofei Chen, Libing Zhou, Tuan Guo

**Affiliations:** ^1^ College of Physics & Optoelectronic Engineering Jinan University Guangzhou China; ^2^ Guangdong‐Hong Kong‐Macau Institute of CNS Regeneration Jinan University Guangzhou China; ^3^ Department of Orthopedics, The First Affiliated Hospital Jinan University Guangzhou China

**Keywords:** diamond, optoelectronic fiber, photothermal therapy, quantum sensing

## Abstract

The photothermal properties of diamond nitrogen‐vacancy (NV) centers enable simultaneous fluorescence emission and localized heating, yet conventional free‐space quantum systems suffer from limited photothermal efficiency (typically<4°C/mW) and inadequate microwave integration (large in size). We developed a miniaturized quantum photothermal fiber probe utilizing a novel metal/polymer/glass composite optoelectronic fiber that simultaneously guides both lightwave and microwave radiation (at GHz). By integrating a micron‐scale diamond at the fiber tip, the probe achieves enhanced photothermal conversion (13°C/mW, 25°C–120°C range) while providing real‐time self‐monitoring through temperature‐dependent zero‐field splitting measurements with 0.2°C thermal resolution at the micrometer scale. This mass‐producible platform represents a new class of biocompatible quantum sensors combining high thermal efficiency and precise temperature feedback, as demonstrated by successful promotion of in vivo spinal cord injury repair through controlled thermal stimulation.

## Graphical abstract

A miniaturized quantum photothermal fiber probe based on a metal/polymer/glass composite optoelectronic fiber that simultaneously guides lightwave and microwave was developed for the first time. The probe achieves enhanced photothermal conversion (13°C/mW, 25°C–120°C range) while providing real‐time self‐monitoring with 0.2°C thermal resolution at the micrometer scale, as demonstrated by successful promotion of in vivo spinal cord injury repair through controlled thermal stimulation.

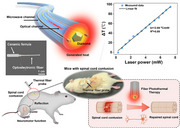

## 1 Introduction

Diamond nitrogen‐vacancy (NV) centers have emerged as quantum sensing platforms, harnessing their optically addressable electron spin states to enable precise measurements characterized by high sensitivity and spatial resolution [1, 2, 3, 4, 5, 6]. These atomic‐scale defects permit detection of diverse physical parameters, including magnetic field [7, 8], temperature [9, 10], pressure [11, 12], and electric field [13, 14], through well‐established quantum protocols such as optically detected magnetic resonance (ODMR), Rabi oscillation, and longitudinal relaxometry. However, the essential laser excitation process inevitably generates localized heating through phonon‐mediated energy conversion [15], creating an intrinsic thermal duality where NV centers function simultaneously as both nanoscale heaters and quantum thermometers. This dual functionality presents unique opportunities for integrated photothermal applications [16, 17]. Recent demonstrations include the work by Sotoma et al., who employed a hybrid diamond nano sensor to measure intracellular thermal conductivities with nanoscale resolution, advancing our understanding of thermal regulation mechanisms in living cells [18]. Despite these advances, conventional free‐space optical coupling architectures impose significant practical constraints, particularly in optically inaccessible environments where waveguide‐integrated solutions become imperative. Moreover, this free‐space coupling approach inherently limits photothermal conversion efficiency (typically< 4°C/mW) [19].

The integration of NV centers with optical fibers presents a compelling strategy for developing miniaturized quantum sensing systems with enhanced portability and functional integration [20, 21, 22]. This architecture capitalizes on guided‐wave photonics to enable in situ quantum measurements within target environments, independent of their optical accessibility, through robust light confinement and propagation in fiber channels. Significant advancements have been made in fiber‐coupled diamond quantum sensors, particularly for magnetometry and thermometry applications [22, 23, 24, 25, 26]. Notable implementations include Graham et al.’s fiber‐optic magnetometer achieving 30 pT/Hz^1/2^ in unshielded environments through comprehensive enhancement methods [27], and Chen et al.’s thermally‐enhanced hybrid sensor employing microfabricated diamond‐PDMS structures to amplify temperature sensitivity by 2.1× via Zeeman energy modulation [20]. Particularly relevant to thermal applications, Duan et al. systematically characterized laser‐induced photonic heating in NV‐embedded microdiamonds and demonstrated fiber‐tip integrated quantum heaters with inherent self‐calibration capabilities [28]. While diamond NV centers have demonstrated highly efficient photothermal effects, their integration into fiber architectures for in vivo phototherapy remains unexplored, representing a critical gap in the development of minimally invasive, high‐precision thermal interventions. Moreover, current implementations remain constrained by their reliance on external microwave antenna systems for spin‐state manipulation, a persistent integration challenge that substantially compromises the miniaturization potential of fiber‐based quantum sensors, particularly in space‐constrained operational environments requiring high spatial efficiency.

In this work, we develop quantum photothermal self‐monitoring fiber probes that integrate dual‐mode photothermal functionality through synergistic integration of optoelectronic fiber architecture with diamond quantum elements. The monolithic device enables simultaneous lightwave and microwave transmission through a single fiber while providing autonomous thermal monitoring during localized heating. The probe achieves laser‐induced temperature elevations of up to 120°C with photothermal conversion efficiency (13°C/mW) and enables real‐time temperature tracking via zero‐field splitting measurements (0.2°C resolution). Systematic validation experiments confirmed operational stability, characterized self‐referenced thermal output in biologically relevant media, and demonstrated therapeutic efficacy in spinal cord injury models. As compared to these classical fiber‐based photothermal probes encompassing configurations such as microfibers modified with photothermal materials or rare‐earth‐doped fibers [29, 30, 31, 32, 33], our quantum fiber probe offers substantial improvements in both photothermal coefficient and miniaturization, overcoming the limitations that impede precise spatial targeting and efficient thermal treatment within microscale biological environments. Ultimately, this work establishes a new paradigm for multifunctional quantum fiber devices with unprecedented spatial integration while bridging the gap between quantum photonic systems and clinical thermal intervention technologies.

To clarify the distinction from existing approaches, a comparison with representative photothermal and fiber‐based thermometry systems is summarized in Table 1. The present work advances the field on three distinct fronts. First, through the synergistic integration of diamond NV centers with a multimaterial optoelectronic fiber that simultaneously guides light and microwave, we achieve a dramatic performance enhancement in photothermal conversion efficiency of 12.99°C/mW (17‐100× higher than existing nanomaterial‐based probes), combined with real‐time self‐monitoring at 0.2°C resolution. This represents the first fiber‐optic device that integrates high‐efficiency heating and precise thermometry within a single monolithic structure. Second, we demonstrate the first in vivo application of a quantum photothermal probe, successfully implanting it in a rat spinal cord injury model and delivering controlled thermal therapy over five days with continuous temperature feedback. Third, we establish a new application paradigm, quantum theragnostic, where a single NV center‐based device simultaneously serves as both a therapeutic heater and a diagnostic thermometer. This dual functionality, intrinsic to NV centers, fundamentally distinguishes our approach from conventional photothermal materials that can only heat. Together, these innovations transform the quantum fiber probe from a laboratory sensor into a clinically deployable therapeutic platform, bridging the gap between quantum photonics and thermal medicine.

**TABLE 1:** Comparison of representative fiber‐based photothermal probes.

Method	Laser‐induced heating (°C/mW)	Temperature detection limit (°C)	Spatial resolution	Ref.
GO‐Au@Ag_2_S nanoscale interface	0.52	None	—	[34]
GO‐Au@Ag_2_S‐VO_2_ nanoscale interface	0.60	None	—	[34]
Er^3+^ /Yb^3+^ co‐doped optical fiber	0.75	None	125µm	[35]
GO‐supported Au NR‐Cu_2‐x_S	0.58	None	—	[36]
Gr/AuNS hybrid nanomaterials	0.74	None	150µm	[37]
ZIF‐90/GO	0.12	None	130µm	[38]
NV center phonon vibration	12.99	0.2	105µm	This work

## 2 Results and Discussion

### 2.1 Quantum Photothermal Principle

**FIGURE 1:**
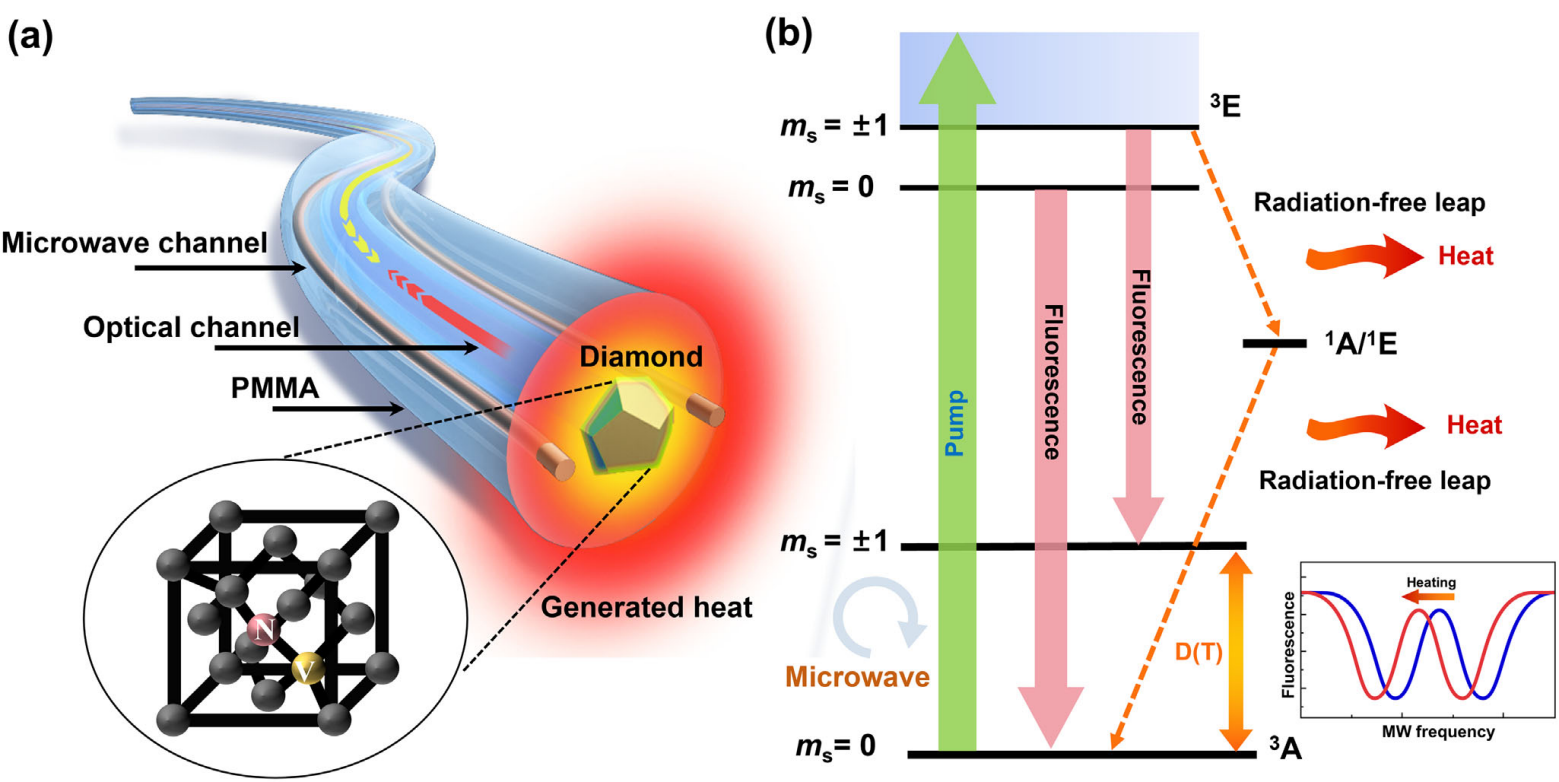
Scheme of quantum photothermal generation over fiber. (a) Schematic diagram of self‐monitoring quantum photothermal fiber probe integrated with multimaterial optoelectronic fiber and diamond NV centers. (b) Energy level diagram of the NV center for illustrations of heat generation and self‐monitoring based on the ODMR method.

The quantum photothermal self‐monitoring fiber integrates diamond NV centers with multimaterial optoelectronic fibers through a thermal drawing process, as schematically depicted in Figure 1a. The composite fiber architecture employs a commercial glass multi‐mode fiber (105 µm diameter) as the optical core, concentrically encapsulated within a poly (methyl methacrylate) (PMMA) cladding.

Two parallel copper electrodes (50 µm diameter) are co‐embedded during fabrication, establishing dual lightwave and microwave transmission channels within a unified fiber structure. The glass core operates as a low‐loss optical waveguide, while the copper electrodes function as microwave transmission lines. The PMMA cladding serves dual structural roles: providing electrical insulation between conductive and optical pathways, and maintaining mechanical stability through viscous encapsulation. This design achieves seamless integration of lightwave and microwave guidance capabilities in a single fiber platform.

The dual photothermal functionality originates from the unique energy level transition characteristics and the temperature‐dependent energy level splitting of the NV center as presented in Figure 1b. The NV center exhibits a spin‐triplet ground state (^3^A_2_) and excited state (^3^E), with intermediate singlet states (^1^A, ^1^E) mediating non‐radiative transitions. Under zero magnetic field, the m_s_ = ±1 sublevels of the ^3^A_2_ state remain degenerate, separated from m_s_ = 0 by the zero‐field splitting (ZFS). Optical excitation using a green laser promotes electrons to the ^3^E state while preserving spin polarization. Direct radiative decay from ^3^E (m_s_ = 0) to ^3^A_2_ (m_s_ = 0) produces broadband fluorescence (550–900 nm) via phonon‐assisted emission. For ^3^E (m_s_ = ±1) states, a fraction decays radiatively, while approximately 30% undergoes intersystem crossing through the ^1^A/^1^E singlet states before relaxing to ^3^A_2_ (m_s_ = 0). This non‐radiative pathway converts ∼23.6% of the excitation energy (0.55 eV/photon) into lattice phonons via vibronic coupling [8], generating localized heating. Concurrently, the temperature‐dependent ZFS shift enables real‐time thermal sensing through ODMR spectral tracking, establishing the dual heating‐sensing mechanism.

The spin dynamics of NV centers in diamond are governed by quantum mechanical interactions between the electronic spin system and its local environment. This behavior can be described by the spin Hamiltonian: [39]

(1) $$H=DS_{Z}^{2}+E\left(S_{x}^{2}-S_{y}^{2}\right)+\gamma_{e}B\cdot S+\sum A_{i}\cdot I_{i}$$

where D = 2.87 GHz (under room temperature) presents the axial ZFS parameter, S = (Sx, Sy, Sz) denotes the electron spin operator, E is the off‐axis ZFS parameter resulting from local strain in the diamond matrix, γ_e_ = 28 GHz/T is the NV gyromagnetic ratio, B is magnetic field, A_i_ denotes the hyperfine coupling tensor between the electron and nuclear spins, and I_i_ is the nuclear spin operator. The first to fourth terms of (1) account for the axial crystal field interactions through temperature‐dependent ZFS, Stark/shift effects from electric fields and lattice strain, Zeeman splitting induced by magnetic fields, and hyperfine interaction with surrounding nuclear spins, respectively. The thermal sensing mechanism exploits the temperature sensitivity of the ZFS parameter D, which arises from thermal lattice expansion modifying the NV center's crystal field environment. The temperature dependence of the zero‐field splitting parameter D is sample‐dependent and influenced by factors such as strain and crystal quality. Therefore, experimental calibration is required for accurate thermometry, and the temperature dependence of the NV center ZFS see supporting information. In this work, we determine an experimental coefficient of −80 kHz/K, which is used for all temperature calculations. The D is quantitatively measured through ODMR spectroscopy. This technique employs simultaneous green laser excitation and frequency‐swept microwave radiation while monitoring spin‐state‐dependent fluorescence intensity. The characteristic resonant dips in the ODMR spectrum correspond to transitions between the m_s_ = 0 and m_s_ = ±1 spin sublevels, whose spectral positions directly reflect the instantaneous D value. By tracking these resonance frequency shifts, we achieve real‐time thermal tracking through the established linear relationship between ΔD and temperature variation. This methodology enables sub‐degree Celsius temperature resolution while maintaining concurrent heating operation through continuous laser excitation.

### 2.2 Quantum Photothermal Fiber

**FIGURE 2:**
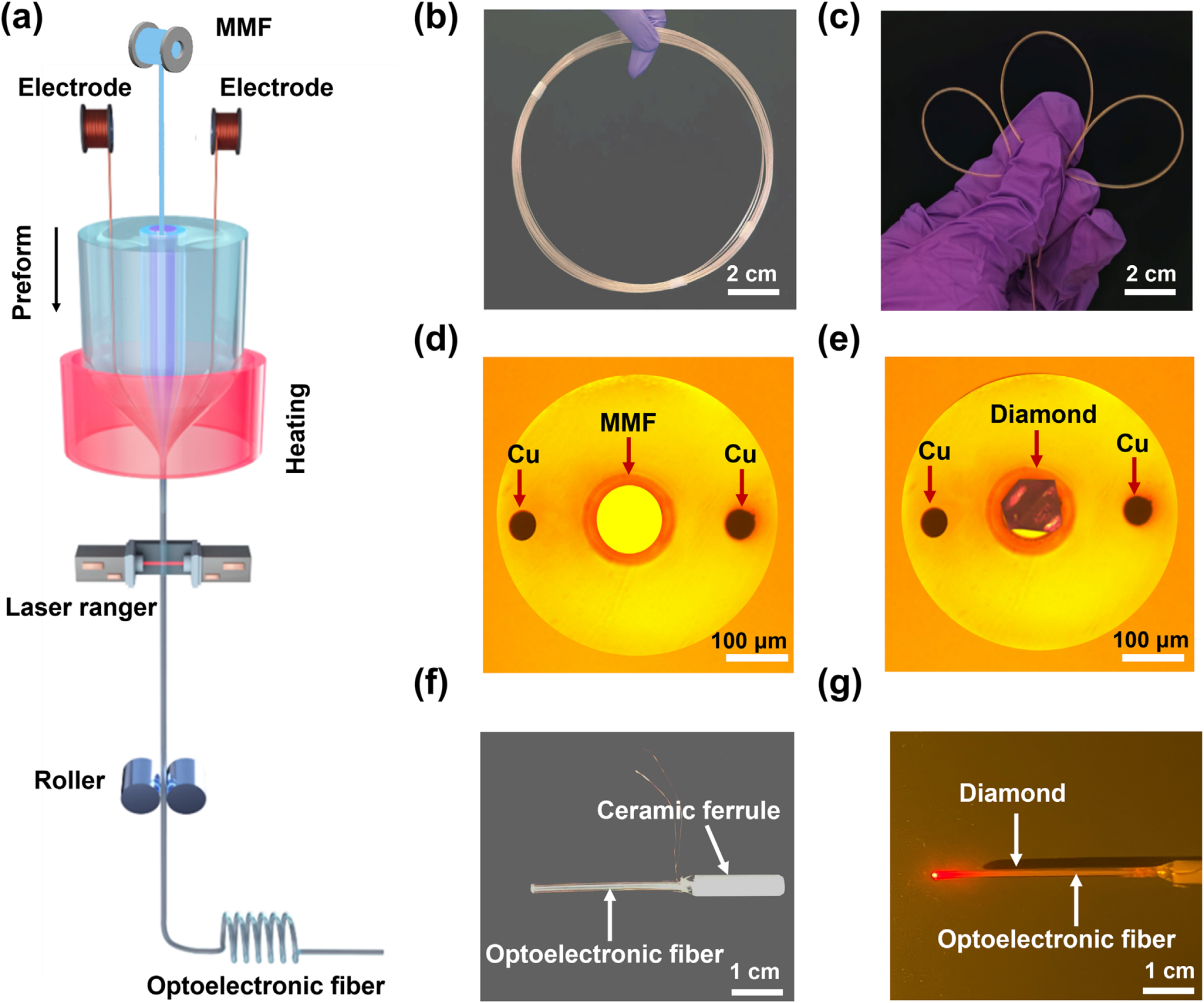
Fabrication and characterization of the quantum photonic thermal fiber. (a) Schematic diagram of the thermal drawing process. (b,c) Images of the drawn optoelectronic fibers. (d) Optical images of cross‐sections of optoelectronic fiber. (e) Optical images of the fiber tip integrated with a diamond. (f) Overall diagram of the prepared all‐fiber device. (g) Optical image of the device under green excitation light illumination with a diamond. A long‐pass red filter placed before the camera reveals intense red fluorescence emission from the diamond during green laser excitation.

The integration of metals, glass fibers, and polymers into a single fiber to create multimaterial optoelectronic fibers at high temperatures is extremely challenging, involving complex softening, extrusion, and co‐solidification processes. In this work, we developed a novel fabrication process for integrating metal electrodes, glass fibers, and polymers to produce optoelectronic fibers, as illustrated in Figure 2a. The resulting fiber architecture maintains coaxial alignment of optical and electrical pathways through viscosity‐matched thermal drawing, achieving precise dimensional control with uniform diameter across continuous lengths (Figure S1). The thermal drawing process was precisely controlled using advanced feedback systems, yielding the large‐area optoelectronic fibers, as shown in Figure 2b. The constructed fiber device exhibits mechanical flexibility. As presented in Figure S2, the stiffness of the optoelectronic fiber is only 1200 N/m with a diameter of 850 µm. It can be manually rolled or woven into various complex configurations, such as a cloverleaf structure, as demonstrated in Figure 2c. Then, the quantum photothermal fiber can be constructed by integrating diamond NV centers on the fiber tip. One end of the fiber was inserted into a ceramic ferrule and secured with UV‐curable adhesive to enable compatibility with conventional fiber connections. Following fabrication, a diamond (∼105 µm diameter) with NV centers was integrated at the fiber tip to construct the quantum device. Microscopic examination of the fiber end‐faces without and with diamond is presented in Figure 2d,e, respectively, which confirms the structural integrity of the device. These optoelectronic fibers enable simultaneous lightwave and microwave transmission for NV center excitation, manipulation, and fluorescence collection (Figure 2f). Furthermore, the diameter of the optoelectronic fiber has almost no effect on its transmittance, as shown in Figure S4, confirming superior light‐guiding capabilities. As shown in Figure S5, the optoelectronic fiber without diamond can effectively transmit 532 nm green laser light and the transmission photo of 635 nm laser light over a long fiber length. While on the device under green excitation light illumination with diamond, intense red fluorescence emission from the diamond can be observed when a long‐pass red filter is placed before the camera (Figure 2g). The measured reflection coefficient S11 of −14.63 dB at ∼2.87 GHz indicates efficient microwave coupling and loading into the transmission structure (Figures S6 and S7). Transmission spectra analysis shows optical transmittance exceeding 98% across fiber lengths of 0–60 cm (Figure S3), indicating that the thermal drawing process preserves the intrinsic optical properties of the glass core without introducing additional loss. To confirm that the diamond NV centers were successfully integrated and could be optically addressed through the fiber probe, we measured the fluorescence spectrum using the complete fiber‐optic system (Figure S8). A 532 nm laser was coupled into the fiber, and the returning fluorescence was collected through a circulator to an optical analyzer spectrometer. The clear NV center signature in the spectrum verified both the quality of the diamond and the functionality of the fiber‐based fluorescence collection. Building on this confirmation, we then quantified the fluorescence collection efficiency of the system, as described in the test system below. The photoluminescence spectrum of diamond under 532 nm laser excitation is presented in Figure S8, and it exhibits a characteristic zero‐phonon line (ZPL) at approximately 637 nm, corresponding to the characteristic of NV^−^ centers in diamond. The final optoelectronic fiber used for all experiments had a diameter of 850 µm, as shown in Figure S2a.

### 2.3 Quantum Photothermal Generation and Monitoring

**FIGURE 3:**
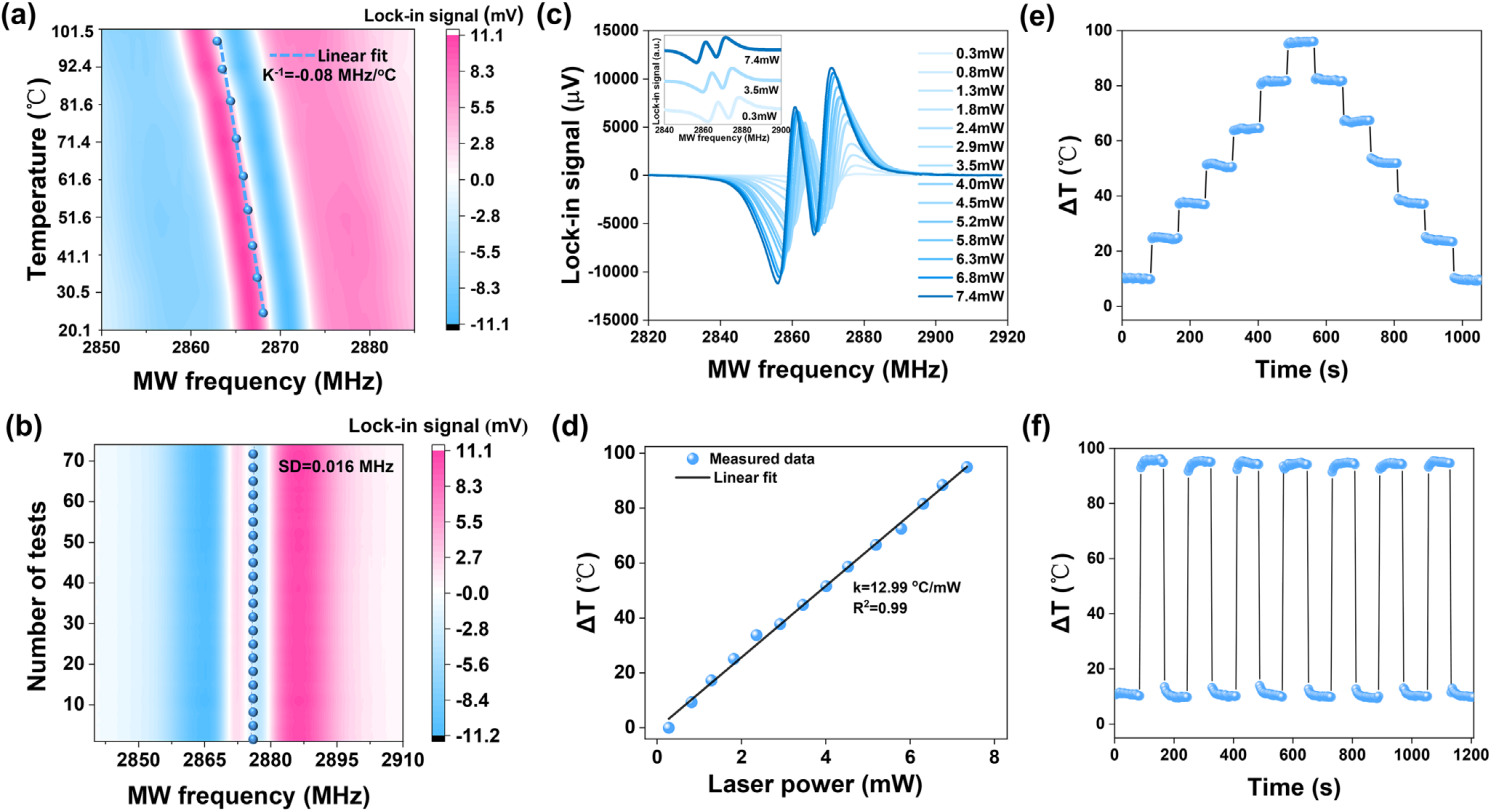
Thermal response and heat generation performance of the quantum photonic fiber. (a) ODMR spectra under different ambient temperatures, and temperature‐dependent shift of the center frequency (dotted line). (b) Noise‐floor characterization results. (c) ODMR spectra under different laser powers. (d) Relationship between laser power and temperature change. (e) Dynamic change of temperature with the step rise of laser power. (f) Results of repeatability test on temperature variation via periodic laser power modulation between 0.8 and 7.4 mW.

The thermal response of the quantum photonic thermal fiber system to the variations in ambient temperature was first characterized, and the measurement system architecture is presented in Figure S9. Initial system calibration quantified the temperature‐dependent ODMR spectral shift, as demonstrated in Figure 3a. Progressive heating induces a systematic shift of the ODMR spectrum to lower frequency, arising from the thermal contraction of the NV center's crystal field that reduces the ZFS parameter D. Linear regression analysis of the central resonance frequencies vs. temperature yields a sensitivity coefficient of −80 kHz/°C, consistent with the intrinsic temperature‐dependent ZFS coefficient of −74 kHz/°C with minor discrepancies attributable to local strain variations in the diamond lattice. To determine the intrinsic thermal resolution, we performed noise‐floor characterization under stabilized thermal conditions. Statistical analysis of 74 consecutive ODMR acquisitions revealed a resonance frequency standard deviation of 16 kHz, corresponding to a temperature detection limit of 0.2°C, as shown in Figure 3b. This resolution can be further enhanced through active thermal stabilization of the measurement environment and laser intensity stabilization. The thermal resolution of the NV‐based thermometer is given by $\delta T=\Gamma /(C\cdot |dD/dT|\cdot \sqrt{N})$, linking sensitivity to linewidth, contrast, and photon count (see Supporting Information for details). In practice, the resolution is influenced by multiple noise sources, including photon shot noise, laser intensity fluctuations, environmental thermal drift, and detector noise. Under stabilized conditions, the residual temperature fluctuation is below 0.05°C, supporting the experimentally measured resolution of 0.2°C.

We systematically investigated laser power effects (0.3–7.4 mW) on the quantum photonic thermal fiber system's ODMR characteristics. Figure 3c demonstrates two concurrent power‐dependent phenomena: progressive low‐frequency shifting of the ODMR spectrum, and enhanced signal contrast through increased spin polarization. The signal‐to‐noise ratio (SNR) of the ODMR measurement exhibits a non‐monotonic dependence on both laser and microwave power due to the interplay between fluorescence intensity, ODMR contrast, and power broadening (Figures S10 and S11). Applying our calibrated thermal response coefficient (−80 kHz/°C), we established direct correspondence between incident laser power and localized temperature elevation (Figure 3d). The system exhibits linear photothermal conversion behavior, achieving the maximum temperature rise of 95°C at 7.4 mW illumination with 13°C/mW efficiency. Control experiments confirmed that this temperature rise predominantly originates from the photothermal effect of the diamond NV centers, rather than heating from the microwave, optical fiber itself, or the UV‐curable adhesive used during fabrication (Figure S12). To evaluate operational robustness and thermal cycling performance, we implemented a characterization protocol for the quantum photonic thermal fiber system. The testing sequence comprised in two forms: discrete power cycling and alternating power cycling. In the former, stepwise laser modulation from 0.8 to 7.4 mW in six increments, followed by reverse‐sequence decrement, with continuous ODMR monitoring (20 spectra/level) for temperature extraction. Figure 3e demonstrates dynamic response and measurement reproducibility across the operational range. The alternating power cycling test was conducted with seven transitions between 0.8 and 7.4 mW to evaluate the system's transient response and thermal hysteresis characteristics. The results shown in Figure 3f reveal stable thermal regulation with negligible hysteresis during maximum power operation. The heating and cooling time constants are 1.32 and 1.38 s, respectively, averaged over seven repeated heating‐cooling cycles (Figures S13 and S14). Transient COMSOL simulations further show that at a position 100 µm away from the diamond, the temperature increases by 65°Cwithin 0.63 s, indicating rapid localized photothermal heating near the diamond tip (Figure S15). The slightly faster simulated response compared with the experiment is attributed to simplified boundary conditions in the numerical model, as well as experimental factors such as finite photothermal generation dynamics, instrumental response time, and additional thermal resistances in the device structure. Overall, both results confirm the fast thermal response and localized heat generation of the device. To further understand heat dissipation, COMSOL Multiphysics simulations were performed. It is indicated that the heat generation is strongly localized at the diamond tip, while the subsequent cooling process is dominated by thermal diffusion (Figure S16). These results validate the system's suitability for clinical thermal intervention protocols requiring precise temperature modulation under dynamic optical excitation conditions.

### 2.4 In Vitro Tissue Photothermal Ablation

First, we validated the biothermal regulation capability of the fiber probe through in vitro ablation experiments using chicken breast tissue, a process requiring high photothermal efficiency and substantial temperature elevation. Infrared thermographic imaging of the fiber probe in air showed laser power‐dependent heating profiles, yielding a photothermal conversion efficiency of 11.1°C/mW (Figure S17a), which is lower than the value obtained from NV‐based thermometry 13°C/mW (Figure 3d). This difference arises from the measurement location and thermal environment. The NV sensor measures the local temperature at the heat source, whereas infrared thermography measures a spatially averaged surface temperature affected by heat diffusion. In addition, increased heat dissipation in tissue leads to lower apparent efficiency. Despite these differences, all measurements show a linear temperature‐power relationship. The differences arise mainly from the measurement location and thermal environment. The NV‐diamond sensor directly measures the temperature at the fiber tip where the photothermal heating is generated. In contrast, infrared thermography measures the surface temperature of the probe, which is spatially averaged and affected by heat diffusion. As a result, the temperature measured by infrared imaging is generally lower than the local hotspot temperature detected by the diamond sensor. After inserting the probe into the tissue, progressive increases in laser power induced localized temperature rises at the fiber–tissue interface reaching 25.6°C, 34.2°C, 47.7°C, and 69.1°C (Figure S17b). Photothermal response analysis revealed a robust linear correlation between laser power and thermal output, yielding a tissue‐specific conversion efficiency of 6.1°C/mW. The reduced slope compared to air‐environment testing originates from enhanced thermal dissipation in biological media. Notably, the system achieves ablative temperatures (>60°C) at 7.4 mW input, a 27‐fold improvement in photothermal conversion efficiency over conventional laser therapies requiring 200 mW for moderate tumor heating [36]. This breakthrough stems from synergistic NV‐mediated energy localization and diamond‐enabled thermal management. The integrated self‐monitoring capability eliminates external thermometry needs while ensuring ±0.2°C regulation precision. Such performance advances position this technology as a platform for precision thermal medicine, particularly in oncology, where localized hyperthermia demands micron‐scale spatial control and real‐time feedback. The demonstrated combination of ultralow‐power operation, embedded diagnostics, and clinical‐grade safety metrics opens new frontiers in minimally invasive thermal therapies.

### 2.5 In Vivo Spinal Cord Injury Repair

Hyperthermia has emerged as a promising therapeutic approach for spinal cord injury (SCI) repair, leveraging localized heat delivery to modulate cellular responses and promote tissue regeneration [40]. The optical fiber hyperthermia technique involves the precise application of thermal energy via implanted fibers, which can mitigate secondary SCI and promote the recovery of sensorimotor function. The minimally invasive nature of optical fiber hyperthermia, combined with its ability to provide real‐time temperature monitoring, positions it as a viable option for clinical translation in SCI treatment. Building upon the quantum photothermal self‐monitoring fiber probe's demonstrated performance, we have verified its application in the

**FIGURE 4:**
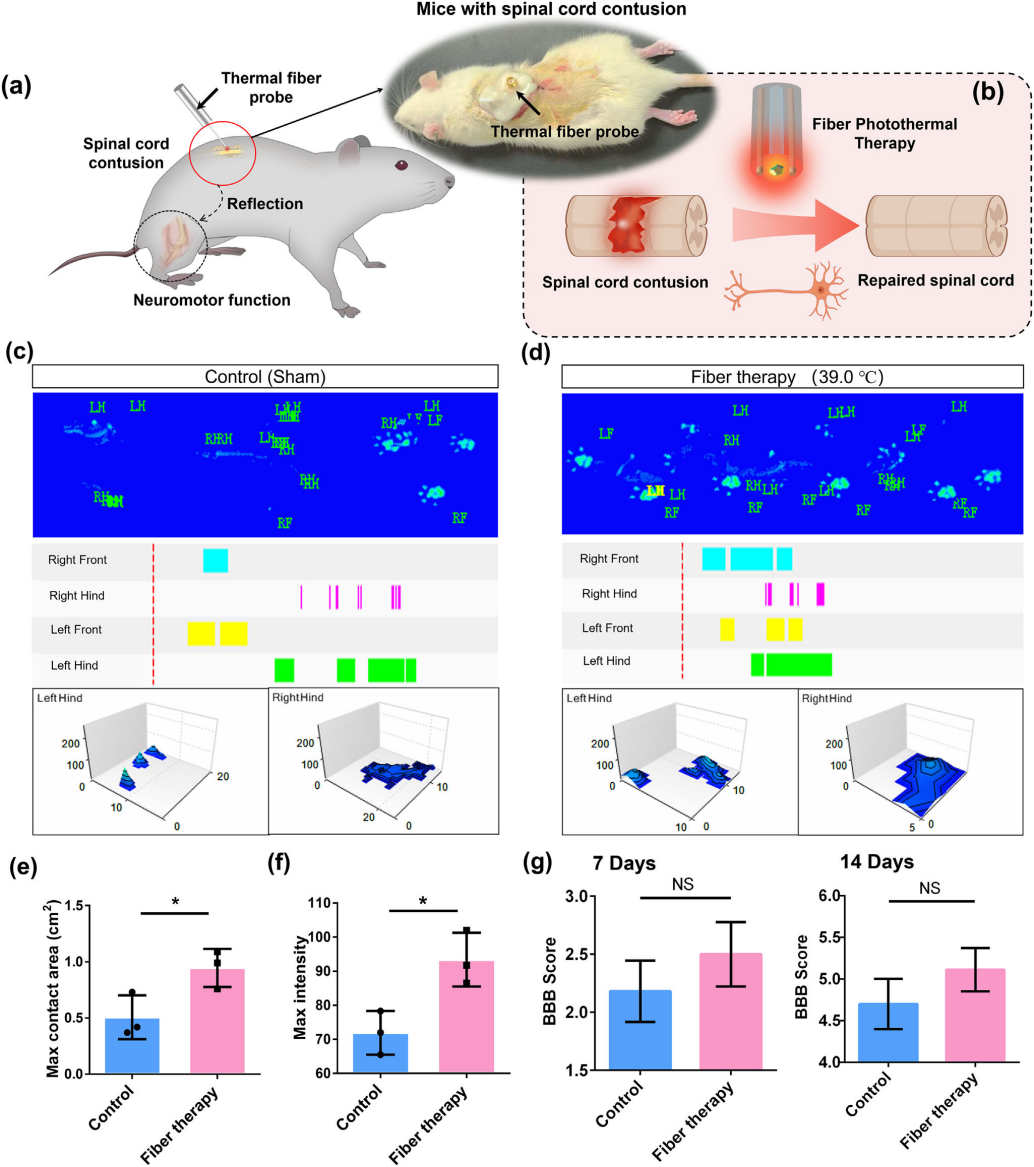
Application of the quantum thermal fiber probe for hindlimb locomotor function repair after spinal cord injury. (a,b) Schematic diagram of the fiber implantation in SCI rats. The quantum fiber probe was implanted on the surface of the spinal cord (T10 segment) to treat SCI. The treatment promoted neurological functional recovery after spinal cord contusion, primarily manifested as improvement in hindlimb motor function. (c–f) CatWalk gait analysis at 2 weeks post‐SCI showed that the fiber therapy group exhibited more regular hindlimb footprints and higher paw pressure (reflecting improved weight‐bearing capacity) compared to the control (Sham) group. (g) BBB scores within 2 weeks post‐SCI appeared higher in the fiber therapy group than in the control (Sham) group. Control (sham) group: animals implanted with the fiber probe without laser irradiation. The data represent repeated measurements from a single animal and therefore illustrate qualitative trends rather than statistically validated results.

Thermal repair treatment of spinal cord injuries. The functionality and longevity of the fiber is assessed by performing a chronic implantation of the fiber into the spinal cord injury site in rats, as presented in Figure 4a and Figure S18. Following the induction of spinal cord injury, experimental subjects received daily fiber‐mediated thermal therapy administered continuously over a five‐day treatment period. To evaluate the therapeutic efficacy of this intervention, longitudinal monitoring of motor function recovery was conducted through systematic behavioral assessments performed at predetermined postoperative intervals (Figure 4b). Quantitative analysis of locomotor performance was achieved by employing two well‐validated measurement systems: the automated CatWalk XT gait analysis platform for detailed kinematic evaluation and the standardized Basso–Beattie–Bresnahan (BBB) locomotor rating scale for functional scoring. As demonstrated in Figure 4c,d, comparative analysis between treatment groups revealed several noteworthy findings. Control animals were implanted with the fiber probe but did not receive laser irradiation, corresponding to a sham‐treatment group (Control). The cohort receiving thermal therapy exhibited a discernible increase in mean plantar contact area during ambulation, accompanied by a corresponding elevation in peak vertical force measurements. These quantitative gait parameters collectively suggested enhanced weight‐bearing capacity and improved locomotor coordination in the intervention group (Figure 4e,f). These results represent preliminary observations from a single subject and are intended to illustrate a qualitative trend rather than provide statistically validated conclusions. Furthermore, while BBB scoring data did not reach conventional thresholds of statistical significance, the consistent directional trend across all evaluation timepoints (days 7 and 14 post‐injury) may indicate a potentially meaningful treatment effect (Figure 4g). BBB scores within 2 weeks post‐SCI appeared higher in the fiber therapy group than in the control (sham) group; however, these results represent observations from a single subject and therefore indicate only a qualitative trend.

These multimodal assessment results, encompassing both automated gait analysis and expert behavioral scoring, provide converging evidence that targeted thermal modulation through fiber‐optic delivery may confer measurable benefits in the context of post‐traumatic spinal cord recovery. The observed improvements in biomechanical parameters, particularly the increased contact area and force generation capacity, strongly suggest that the intervention group achieved superior functional restoration compared to untreated controls. This therapeutic advantage was further supported by the positive, though not statistically significant, trend in BBB locomotor scores, which may reflect early‐stage recovery processes not captured by the current measurement paradigm. Future studies with larger cohorts will be necessary to systematically evaluate therapeutic efficacy and establish statistical significance.

To contextualize device performance, we compare key metrics with clinically relevant requirements for spinal cord injury thermal therapy (Table 2). Overall, this analysis demonstrates that our device is not merely a laboratory prototype with impressive specifications, but a clinically informed platform designed to meet the specific requirements of SCI thermal therapy. The proposed fiber probe enables microscale photothermal heating with simultaneous in situ temperature readout, which distinguishes it from conventional photothermal systems relying on external thermometry. The localized heating originates from the diamond element at the fiber tip, and the relatively low thermal conductivity of the surrounding media allows the formation of a confined thermal region on the order of 100–200 µm. Despite these advantages, several practical limitations should be considered. The current fiber diameter (850 µm) may limit applications requiring minimal invasiveness. This constraint arises primarily from fabrication and mechanical considerations in the present design rather than fundamental limitations. Further miniaturization is feasible and represents an important direction for future work.

**TABLE 2:** Clinical requirements for SCI thermal therapy vs. device performance [41, 42, 43, 44, 45, 46, 47, 48, 49, 50].

Clinical requirement	Target range/specification	Clinical rationale	Our device performance
Temperature range for therapeutic effect	39°C–43°C (mild hyperthermia) >60°C (thermal ablation)	Mild hyperthermia promotes neuroprotection and regeneration; ablation temperatures for tumor treatment	25°C–120°C 36°C–40°C with <0.5°C precision 69.1°C achieved in tissue
Temperature control precision	±0.5°C for mild hyperthermia	Prevents thermal damage to spinal cord tissue; avoids under‐treatment	<0.5°C demonstrated
Spatial precision of heating	<1 mm	Confines thermal effect to the injury site; spares surrounding healthy tissue	Micron‐scale diamond at fiber tip (105 µm × 105 µm)
Real‐time temperature monitoring	Essential for safety	Enables closed‐loop control; prevents overheating	0.2°C resolution; continuous ODMR‐based monitoring
Probe diameter nanomaterials	<1 mm for the spinal cord	Minimizes tissue displacement and inflammatory response	850 µm (current) 50–100 µm achievable with optimization
Biocompatibility	ISO 10993 standards	Required for clinical translation	Materials: glass, PMMA, copper (generally biocompatible);
Treatment duration per session	10–30 min	Clinically practical treatment time	Stable performance over 20 min
Multiple treatment sessions	5–7 daily sessions	Typical SCI rehabilitation protocol	Stable over a 5‐day treatment course

## 3 Conclusion

This study presents a quantum photonic thermal fiber system that integrates diamond NV center‐based photothermal heating and self‐monitoring with high precision. The system demonstrates a linear temperature‐dependent spectral response with sensitivity of −80 kHz/°C, allowing real‐time temperature detection at 0.2°C resolution. It achieves efficient photothermal conversion, reaching 95°C at 7.4 mW laser power (13°C/mW in air) with minimal hysteresis and high dynamic accuracy. In vitro, the probe attained ablative temperatures (>60°C) at ultralow power, showing 27‐fold higher efficiency than conventional hyperthermia techniques. In a rat spinal cord injury model, the implanted fiber provided daily thermal therapy over five days, resulting in significantly improved motor function, weight‐bearing, and coordinated locomotion. The technology enables real‐time feedback and precise thermal intervention without external thermometry, offering a platform for minimally invasive thermal medicine in applications such as oncology and neurorepair.

## 4 Experimental Section

### 4.1 Fabrication of Optoelectronic Fibers

**FIGURE 5:**
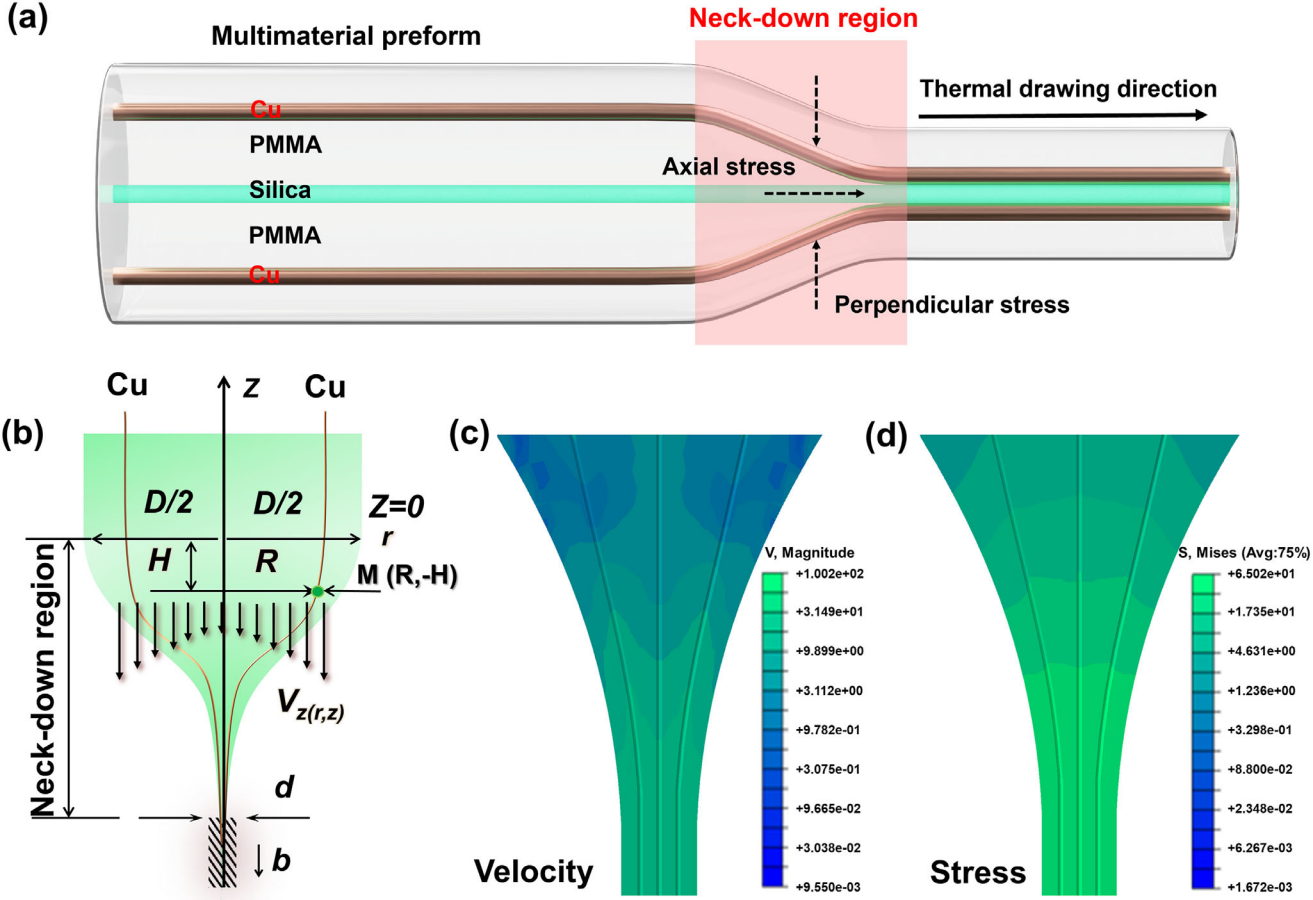
Theoretical analysis of the multimaterial integration process. (a) The snap spot on the neck‐down region during the fiber drawing process. (b) The shear stress analyses of metal and glass during the fiber drawing process. (c) The fluid velocity distribution in the neck‐down region. (d) The stress distribution of the fiber in the neck‐down region.

To fabricate high‐quality metal‐glass‐polymer multimaterial optoelectronic fibers, we first investigated the stress distribution among the constituent materials during thermal drawing. In this process, the selected metal electrodes and glass fibers maintain their structural integrity as their melting points exceed the drawing temperature. Consequently, while the polymer exists in a viscous supercooled liquid state, both the glass fibers and metal electrodes remain solid throughout the drawing process. We hypothesize that the flow of the viscous supercooled liquid polymer induces shear stress on the metal electrodes and glass fibers, as illustrated in Figure 5a.

Figure 5b illustrates the shear stress distribution τ(z,r) exerted on both the metal electrode and glass fiber by the viscous polymer. Under the assumption that the upper boundaries of the metal electrodes and glass fiber remain fixed within the polymer matrix, and given the velocity field distribution Vz(r,z) of the viscous polymer flow, we analyze the resulting shear stress using Newton's law of internal friction [51]. Specifically, the shear stress induced by polymer flow at point M(R,‐H) on the metal electrode surface is expressed as:

(2) $$\tau_{1}=\mu \cdot \frac{\partial V_{z}}{\partial r}{|}_{r=R}$$

where µ is the viscosity of the polymer cladding, R is the radial distance from point M to the *z*‐axis, and H is the axial distance from point M to the *r*‐axis. Similarly, the shear stress at the central glass fiber is:

(3) $$\tau_{2}=\mu \cdot \frac{\partial V_{z}}{\partial r}{|}_{r=0}$$

The pulling forces exerted on the metal electrode (F_1_) and glass fiber (F_2_) are then given by:

(4) $$F_{1}=\int_{0}^{-H}\mu \cdot \frac{\partial V_{z}}{\partial r}{|}_{r=R}dz$$

(5) $$F_{2}=\int_{0}^{-H}\mu \cdot \frac{\partial V_{z}}{\partial r}{|}_{r=0}\mathrm{dz}$$

To determine the pulling force, the radial velocity gradients on the metal electrode $\frac{\partial V_{z}}{\partial r}{|}_{r=R}$ and glass fiber $\frac{\partial V_{z}}{\partial r}{|}_{r=0}$ must be calculated. For this purpose, the viscous polymer flow is divided into two regions. The velocity distribution in the upper region is:

(6) $$V_{z1\left(r\right)}=kr\left(r-P/2\right)\;\left(r>d\right)$$

where k is a constant coefficient, and P is the diameter of the initial preform. The velocity distribution in the lower region is:

(7) $$V_{z2\left(r\right)}=S\;\left(0\leq r\leq d\right)$$

where S is the fiber drawing speed, and d is the final fiber diameter. Applying the law of flow conservation [52], the following relationship is obtained:

(8) $$\int_{0}^{P/2}2\pi rV_{z}dr=Q=\frac{\pi }{4}\;Sd^{2}$$

where Q is the flowing volume of the viscous flow polymer. Combining these equations, the pulling force on the metal electrode is derived as:

(9) $$F_{1}=\frac{\mu SHd^{2}\left(48R-12P\right)}{P^{4}}\;$$

Similarly, the force on the central glass fiber is:

(10) $$F_{2}=\frac{12\mu SHd^{2}}{P^{3}}\;$$

The results demonstrate that the tension exerted on both the metal electrodes and glass fiber exhibits a linear dependence on the polymer viscosity (µ), the axial distance (H), the fiber drawing speed (S), and the square of the fiber diameter (d^2^). Conversely, the force acting on the central glass fiber shows an inverse proportionality to the cube of the preform diameter (P^3^). These findings establish a theoretical framework for the rational design of drawing parameters to fabricate high‐quality optoelectronic fibers.

To elucidate the breaking mechanism, finite element analysis (FEA) was performed to investigate the flow velocity and internal stress distributions during the multimaterial integration process. In the simulation, two copper filaments (50 µm diameter) served as metal electrodes, while a 30 mm diameter PMMA cladding was modeled, and the other key parameters were presented in Supplementary Information. The fiber drawing temperature was maintained at 265°C. Figure 5c presents the flow velocity distribution of the viscous glass system, revealing that the maximum velocity occurs at the tip of the neck‐down region and gradually decreases toward the top. Figure 5d displays the internal stress distribution in both the metal filaments and glass. Notably, stress concentration in the copper filaments is most pronounced at the neck‐down region tip. Consequently, when the deformation rate of the copper filaments cannot match that of the glass and the induced shear stress exceeds the copper's yield stress, fracture is likely to occur in this region. These simulation results align with experimental observations, where filament breakage predominantly occurs in the neck‐down region during thermal drawing.

The fabrication of optoelectronic fibers proceeds through two sequential phases: preform fabrication and thermal drawing. Our process employs PMMA as the primary cladding material, selected for its optimal viscosity‐temperature characteristics during drawing. The preform assembly begins with a cylindrical PMMA rod (Ø30 mm × 120 mm) precision‐machined to create a central longitudinal bore (Ø8 mm × 120 mm) for optical core integration, and two parallel ancillary channels (Ø3 mm × 120 mm) for electrode embedding. This structured preform undergoes thermal processing in a fiber drawing tower under controlled temperature and tension parameters.

During the thermal drawing process, the preform was fed into the furnace at a rate of 1 mm/min and drawn at a speed of 2 m/min under controlled conditions. Two copper electrodes, each with a diameter of 50 µm, were continuously fed into the two smaller holes of the preform, while a commercial multimodal fiber with a diameter of 105 µm was continuously fed into the central hole.

### 4.2 Test System

The ODMR‐based test system architecture is shown in Figure S9. A 532 nm laser (MGL‐FN‐532, CNI Optoelectronics) couples into the quantum photonic thermal fiber system via a fiber circulator (WMC3L1S, Thorlabs). The diamond‐integrated fiber tip generates red fluorescence, with a portion back‐coupled through the circulator for detection. The fluorescence signal undergoes spectral purification using a long‐pass filter (FELH0600, Thorlabs, >600 nm cutoff) before intensity measurement via an avalanche photodiode (APD410A/M, Thorlabs). To quantify the end‐to‐end fluorescence collection efficiency of the complete fiber probe system, we measured the fluorescence power at the detector under controlled excitation conditions. Using the same fiber‐optic configuration as for the spectral measurement, we coupled 7.4 mW of 532 nm laser power into the fiber and measured the fluorescence power reaching the avalanche photodiode after passing through the 600 nm long‐pass filter and fiber circulator. The measured fluorescence power was 2.15 µW. The end‐to‐end fluorescence collection efficiency η_collection_ is defined as: η_collection_ = P_fluo,detected_ / P_laser,input_ =  0.029%.

Microwave delivery employs the fiber‐embedded copper electrode as a radiative antenna, fed by a microwave source (SMB100A, Rohde & Schwarz) and amplified (ZHL‐16W‐43‐S+, Mini‐Circuits) to drive NV spin transitions. A 50 Ω‐terminated isolator protects the amplifier from reflected power while maintaining impedance matching. To enhance signal‐to‐noise ratio and acquisition speed, we implement phase‐sensitive detection through frequency‐modulated microwave excitation synchronized with a lock‐in amplifier (OE1022D, Sine Scientific Instruments). The APD output connects to the signal input channel, enabling real‐time demodulation of ODMR spectral features.

### 4.3 Spinal Cord Injury Model and Fiber Implantation

All experiments involving animals were approved by the Jinan University Institutional Animal Care and Use Committee. Following anesthesia, mice were positioned in a prone position on the surgical platform. The dorsal fur was shaved, and the skin was disinfected with iodophor swabs. A laminectomy was performed at the T10 vertebral level to expose the underlying spinal cord tissue. Subsequently, a controlled spinal cord injury was induced using a precision impactor. Upon completion of the injury procedure, an optical fiber device was stereotaxically implanted and secured with dental acrylic cement. In contrast to photothermal ablation therapy, spinal cord thermal repair requires precise temperature modulation within a narrow range. To demonstrate the capability of our fiber probe in meeting this requirement, relevant tests were conducted. The results indicated that, as shown in Figure S19, by precisely controlling the incident laser power, the probe achieved accurate regulation within the range of 36°C–40°C with a precision better than 0.5°C and repeatability. Although this level of control precision may not represent the optimal achievable performance, it meets the requirements for thermal therapy in spinal cord injury.

### 4.4 Catwalk Tests

Prior to testing, animals were acclimated to a dark and quiet environment for 2 h. Each mouse was allowed to traverse a glass panel a minimum of three times, with a camera positioned beneath the panel recording their footprints. The recorded data were then automatically analyzed to assess walking parameters. Coordination during locomotion was evaluated based on the Regularity Index and the maximum paw contact area with the glass surface.

### 4.5 BBB Scoring

The Basso, Beattie, and Bresnahan (BBB) scale is the most widely used method for evaluating hindlimb motor functional recovery in rats following SCI. Postoperative BBB scoring was performed for all rats. To minimize bias, two independent blinded observers, who were not involved in the experimental procedures and were well‐trained in the scoring criteria, conducted the assessments. Each rat was observed for 5 min per session, with three repeated measurements, and the average score was calculated. The final score for each animal represented the mean value from both observers.

In the sham group, rats exhibited slight hindlimb joint movement at 2 h post‐surgery, transient hypoactivity on day 1, and gradual functional recovery by day 3, after which their scores remained stable. In contrast, SCI group rats displayed complete paraplegia at 2 h post‐injury, characterized by loss of hindlimb movement, sensory and defecation dysfunction, and inability to feed voluntarily.

## Author Contributions

Wanjun Li, Ruixiao Hu, Jie Mao, and Jin Wo contributed equally to this work. Minghui Du, Yaofei Chen, Libing Zhou, and Tuan Guo conceived and supervised the project. Wanjun Li, Jie Mao, Liang Chen, Ying Liu, and Zhibo Li fabricated the fiber probes. Wanjun Li, Ruixiao Hu, and Jie Mao characterized and performed the fiber probes. Wanjun Li, Ruixiao Hu, Jie Mao, Wanjun Li, and Yunsong Zhang carried out the thermal treatment experiments in mice. Ying Liu provided guidance on analyzing mouse experimental data. Wanjun Li, Jin Wo, Minghui Du, and Yaofei Chen wrote the paper with discussion from all other authors.

## Conflicts of Interest

The authors declare no conflicts of interest.

## Supporting information




**Supporting File**: advs75733‐sup‐0001‐SuppMat.docx.

## Data Availability

The data that support the findings of this study are available from the corresponding author upon reasonable request.
