# Supplementary material for: Quantum Photothermal Self‐Monitoring Fiber Probes for In Vivo Photothermal Therapy
Source: Adv Sci (Weinh). 2026 May 20:e75733. Online ahead of print. doi: 10.1002/advs.75733 (PMC13335859; doi:10.1002/advs.75733)
Supplement: Supplementary file 1 — Supporting File: advs75733‐sup‐0001‐SuppMat.docx. [file ADVS-9999-e75733-s001.docx]

**Supplementary Information**

**Quantum Photothermal Self-Monitoring Fiber Probes for *In Vivo* Photothermal Therapy**

Wanjun Li^1,a^, Ruixiao Hu^1,a^, Jie Mao^1,a^, Jin Wo^1,b,c^, Yunsong Zhang^b^, Liang Chen^a^, Ying Liu^a^, Zhibo Li^a^, Yongxin Lin^c^, Minghui Du^a,^*, Yaofei Chen ^a,^*, Libing Zhou^b,^ * and Tuan Guo^a,^*

^a^ College of Physics & Optoelectronic Engineering, Jinan University, Guangzhou 510632, P. R. China

^b^ Guangdong-Hongkong-Macau Institute of CNS Regeneration, Key Laboratory of CNS Regeneration (Jinan University)-Ministry of Education, Guangdong Key Laboratory of Non-human Primate Research, Jinan University, Guangzhou 510632, P. R. China

^c^ Department of Orthopedics, The First Affiliated Hospital, Jinan University, Guangzhou 510632, P. R. China

**Finite Element Analysis**

During the simulation, the Young's modulus of the preform made of polymethyl methacrylate (PMMA) is set to 3160 MPa, with a Poisson's ratio of 0.32. The Young's modulus of the copper electrode is 110 GPa, with a Poisson's ratio of 0.32. The Young's modulus of the glass fiber is 76.7 GPa, with a Poisson's ratio of 0.17. The drawing ratio for the optoelectronic fiber is set to 1:50. The stress and flow velocity distributions of the metals, glass fibers, and PMMA are calculated using the following theoretical models:

1）Linear elastic analysis is applied before the material reaches its yield point.

2）Elastic-plastic analysis is performed after the material reaches its yield point.

**Microwave Transmission and Electromagnetic Simulation**

The measured S11 parameter reaches -14.63 dB at ~2.87 GHz, indicating efficient microwave coupling and loading into the transmission structure. HFSS simulations were performed for dielectric constants of 2.3, 2.5, and 2.7. The corresponding maximum magnetic field strengths are 0.136, 0.133, and 0.128 A/m, respectively. The small variation indicates that the microwave field is robust against dielectric variations. In all cases, the magnetic field is concentrated near the transmission line and decays rapidly with distance.

**Theoretical Analysis of Thermal Resolution and Noise Contributions**

The thermal resolution of NV-center-based thermometry is determined by the precision of ODMR frequency measurement. It can be expressed as:^1^

$\delta T=\frac{\delta f}{\mid dD/dT\mid}$ (1)

where $\delta f$ is the uncertainty in frequency determination and $\mid dD/dT\mid$ is the temperature coefficient of the zero-field splitting.

For ODMR-based detection, the frequency uncertainty is governed by the linewidth $\Gamma$, contrast $C$, and signal-to-noise ratio (SNR), leading to:^2^

$\delta f\approx\frac{\Gamma}{C\cdot\mathrm{SNR}}$ (2)

Under shot-noise-limited conditions, the SNR is proportional to the square root of the detected photon number *N*:^3,4^

$\mathrm{SNR}\approx\sqrt{N}$ (3)

Combining these relations yields:

$\delta T=\frac{\Gamma}{C\cdot\mid dD/dT\mid\cdot\sqrt{N}}$ (4)

which defines the intrinsic sensitivity limit.

In practical systems, the total frequency uncertainty arises from multiple noise sources:

$\partial f_{\_total}^{2}=\partial f_{\_shot}^{2}+\partial f_{\_laser}^{2}+\partial f_{\_drift}^{2}+\partial f_{\_elec}^{2}$ (5)

Here, $\partial_{\_shot}$ represents photon shot noise, $\partial_{\_laser}$ arises from laser intensity fluctuations ($\partial P/P$), $\partial_{\_drift}$ is associated with environmental temperature variations, and $\partial_{\_elec}$ corresponds to detector electronic noise. Among these, shot noise defines the fundamental limit and scales as $1/\sqrt{N}$ Laser intensity noise affects the ODMR signal amplitude and contrast, thereby influencing frequency extraction. Environmental thermal drift directly shifts the resonance frequency via $\mid dD/dT\mid\cdot\partial_{\_env}$ and is typically the dominant low-frequency noise source. Electronic noise is generally negligible under sufficient signal levels. The experimentally reported thermal resolution (0.2 °C) is determined from noise-floor characterization under stabilized conditions. These literatures shows that the residual temperature fluctuation is estimated to be below 0.05 °C, providing a quantitative definition of thermal stability. This framework also explains the role of system parameters: reducing linewidth $\Gamma$ (longer T2*), increasing contrast *C*, and enhancing photon collection efficiency (higher N) all improve thermal resolution.^5,6^ However, these parameters are not fully independent, as increasing excitation power may simultaneously increase photon count while broadening the linewidth. Overall, this analysis establishes a quantitative relationship between system parameters, noise sources, and thermal resolution, and provides guidance for further optimization toward the theoretical limit.

**Temperature Dependence of NV Center ZFS**

The thermal sensing mechanism exploits the temperature sensitivity of the ZFS parameter $D$, which arises primarily from electron-phonon interaction. The temperature dependence is well described by the modified Varshni equation:^7^

$E(T)=E_{0}-\frac{AT^{4}}{{(T+B)}^{2}}$ (6)

A modified Varshni formula describes the temperature dependent zero field splitting and zero phonon line of the nitrogen vacancy center in diamond. The shift of energy gaps shows a T^4^ scaling at low temperature and a T^2^ scaling at high temperature.

Due to diamond's exceptionally high Debye temperature ($\Theta_{D}\approx2200\text{ }K$) and very small thermal expansion coefficient ($1.6\times{10}^{-6}\text{ }K^{-1}$), the temperature dependence of NV centers differs from conventional semiconductors and exhibits sample-to-sample variation depending on local strain, nitrogen concentration, and crystal quality. Values reported in the literature typically range from -70 to -80 kHz/K.^8-10^

For accurate thermometry, in-situ experimental calibration is essential. In this work, we experimentally calibrated the temperature response of our specific system. The measured value is:

${\frac{dD}{dT}\mid}_{\mathrm{experimental}}=-80\text{ }kHz\cdot K^{-1}$ (7)

This experimentally determined coefficient is used throughout this work for all temperature calculations.

**Dependence of** **ODMR Signal-to-Noise Ratio on Laser and Microwave Power**

The SNR exhibits a non-monotonic dependence on both laser and microwave power (Fig. S10, S11). At low laser power, increasing excitation enhances fluorescence intensity and improves optical pumping, leading to higher SNR. At higher power, saturation and thermal effects reduce ODMR contrast. Due to the competition among these factors, the SNR curve may exhibit multiple local maxima rather than a simple monotonic trend. A similar behavior is observed for microwave power. Increasing microwave power improves spin driving at low levels, while excessive power leads to linewidth broadening, reducing SNR. These results indicate the presence of optimal operating conditions for ODMR measurements.

**Experimental and Simulation Evaluation of Thermal Response and Thermal Filed Distribution**

To evaluate the temporal response of the device, seven consecutive heating-cooling cycles were experimentally measured. The extracted average heating and cooling time constants are 1.32 s and 1.38 s (Fig. S13, S14), respectively, indicating a rapid and reproducible thermal response. To further analyze the thermal dynamics, transient thermal simulations were performed using COMSOL Multiphysics. The temperature evolution was monitored at a location 100 μm away from the diamond sensor. The simulation shows that the temperature increases by 65 °C within approximately 0.63 s (Fig. S15), indicating fast localized photothermal heating near the diamond tip. The difference between the simulated and experimental heating dynamics mainly arises from factors not fully included in the simplified numerical model. In practice, photothermal heating originates from electronic relaxation processes of the NV centers, which introduce a finite response time for energy conversion. In addition, the instrumental response time of the temperature measurement system can slightly broaden the observed transient signal. Furthermore, the real device involves additional thermal resistances and environmental effects, including polymer encapsulation, interfacial thermal resistance, and convective heat transfer, which can slow down the effective heat dissipation. Spatial temperature distributions were also calculated to analyze the heat dissipation pathway. When the diamond temperature reaches 120 °C, the surrounding temperature remains approximately 100 °C within 500 μm, while it decreases rapidly to about 30 °C at 1000 μm (Fig. S16). This result indicates strong spatial confinement of heat near the diamond tip. Such rapid temporal response combined with microscale thermal confinement is advantageous for precise localized thermal modulation in biomedical applications.


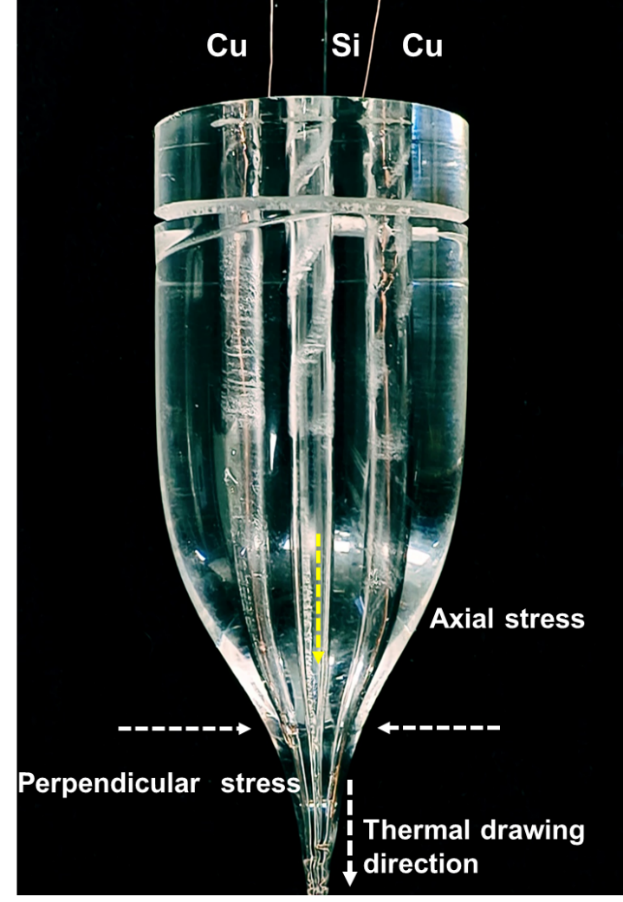


**Figure S1** The image of the remaining macroscopic preform waste after the thermal drawing of multimaterial optoelectronic fiber. The image of the preform used for the thermal drawing of optoelectronic fibers reveals a sophisticated macroscopic blueprint of the final fiber structure. It typically consists of a solid glass core, two copper electrodes precisely positioned within a transparent polymer PMMA cladding. The entire assembly is a meticulously constructed, multi-layered structure where the precise geometric arrangement, material composition, and interfacial cleanliness between these disparate materials are paramount, as they will be scalable reduced in size yet perfectly preserved during the thermal drawing process to form the functional, hair-thin fiber.


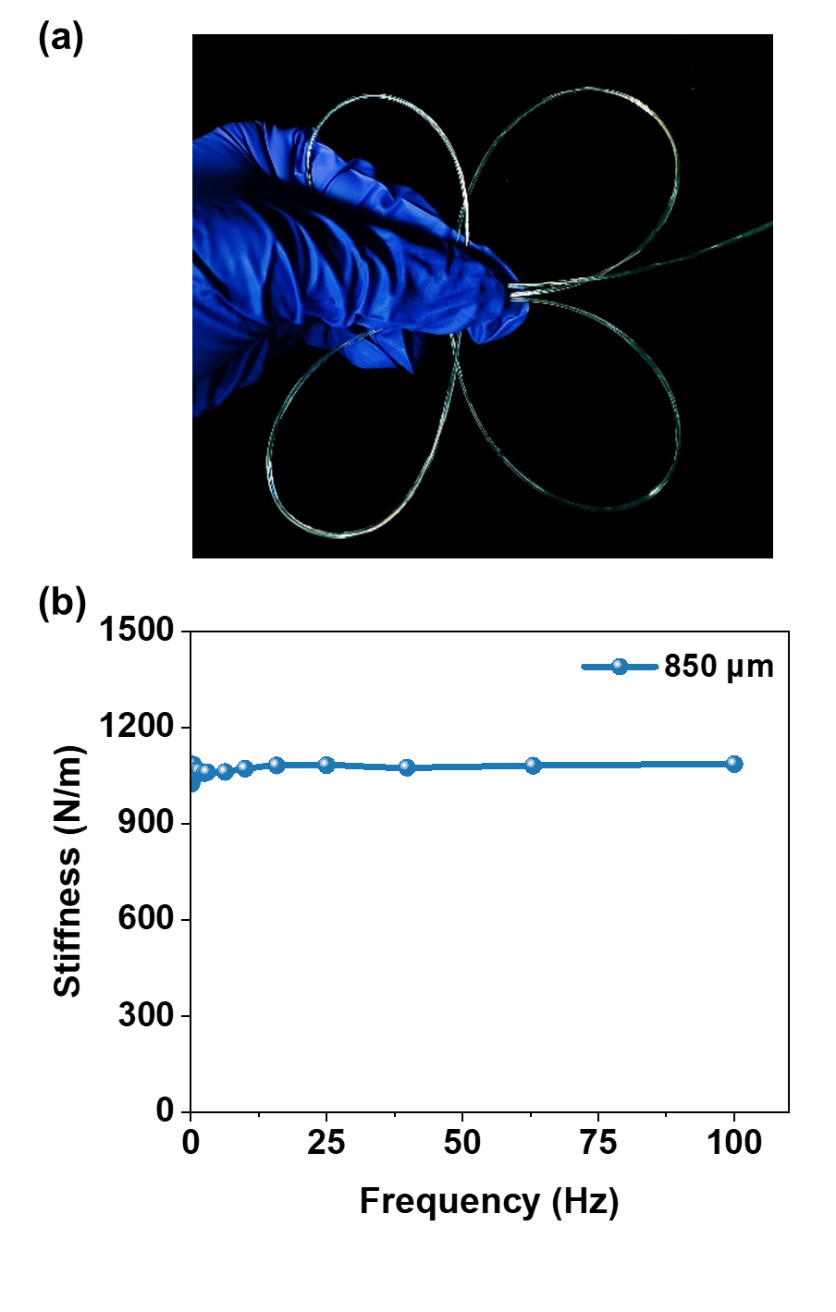


**Figure S2** The photo (a) and mechanical performance (b) of the drawn optoelectronic fiber with a diameter of 850 μm. The constructed fiber exhibits mechanical flexibility, a key characteristic quantified by its remarkably low stiffness of just 1200 N/m despite a substantial diameter of 850 µm. It can be seamlessly rolled, bent, or woven into various complex and stable configurations without sustaining damage, as demonstrated by its formation into intricate shapes like a cloverleaf structure.


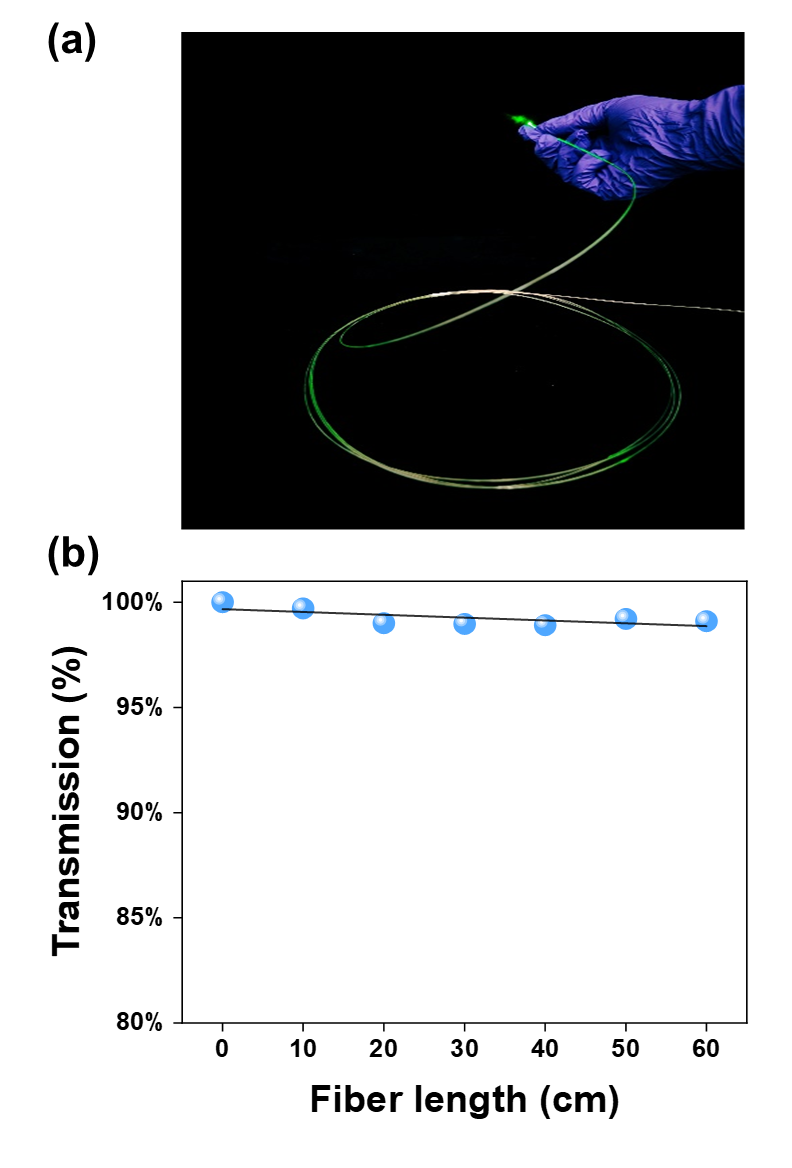


**Figure S3** Process validation. The photo (a) and transmission spectrum (b) of the optoelectronic fiber under 532 nm laser excitation, demonstrating optical performance of the drawn optoelectronic fibers. The results reveal consistently high transmittance exceeding 98% across all the fiber lengths ranging from 0 to 60 cm. This confirm that the multimaterial thermal drawing process does not introduce additional optical loss.


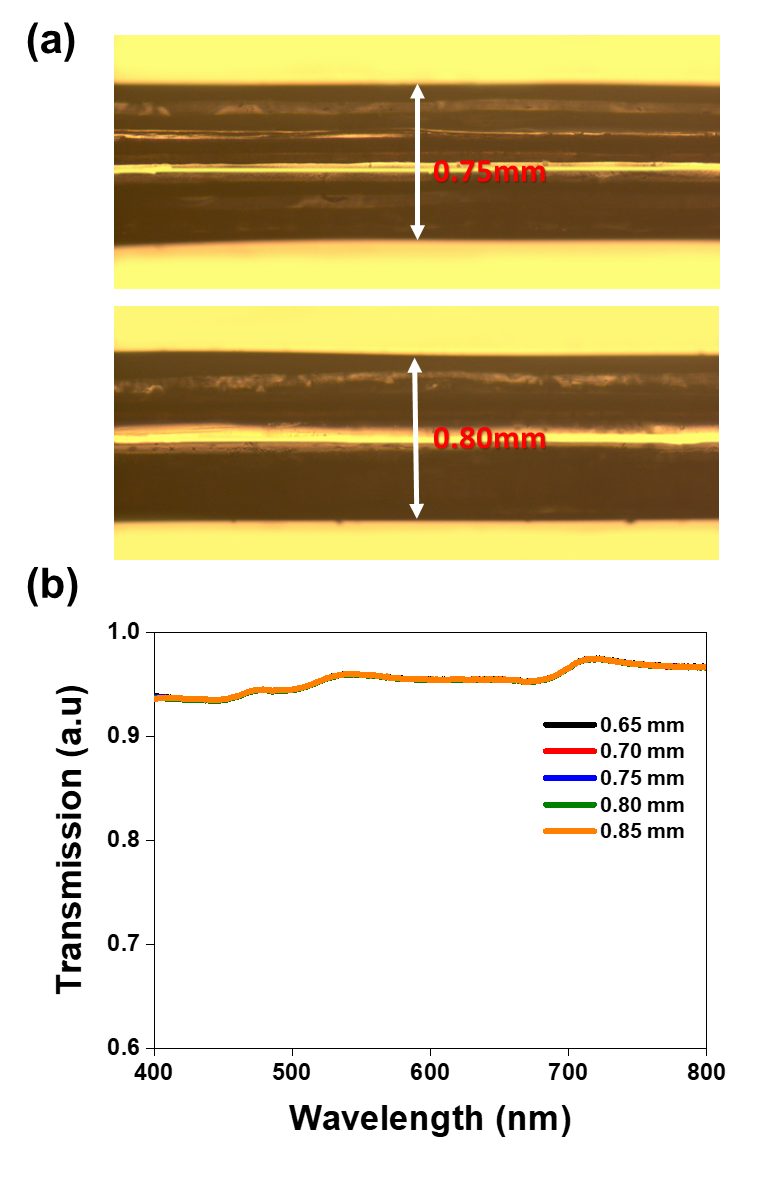


**Figure S4** Diameter independence. The photos (a) and transmission spectrum (b) of optoelectronic fibers with different fiber diameters at the wavelength of 532 nm. The transmission spectrum of the optoelectronic fibers with diameters of 0.65, 0.70, 0.75, 0.80, and 0.85 mm at the 532 nm are nearly identical and exhibit a high degree of overlap. It shows that the diameter of the optoelectronic fiber has almost no effect on its transmittance, confirming superior light-guiding capabilities.


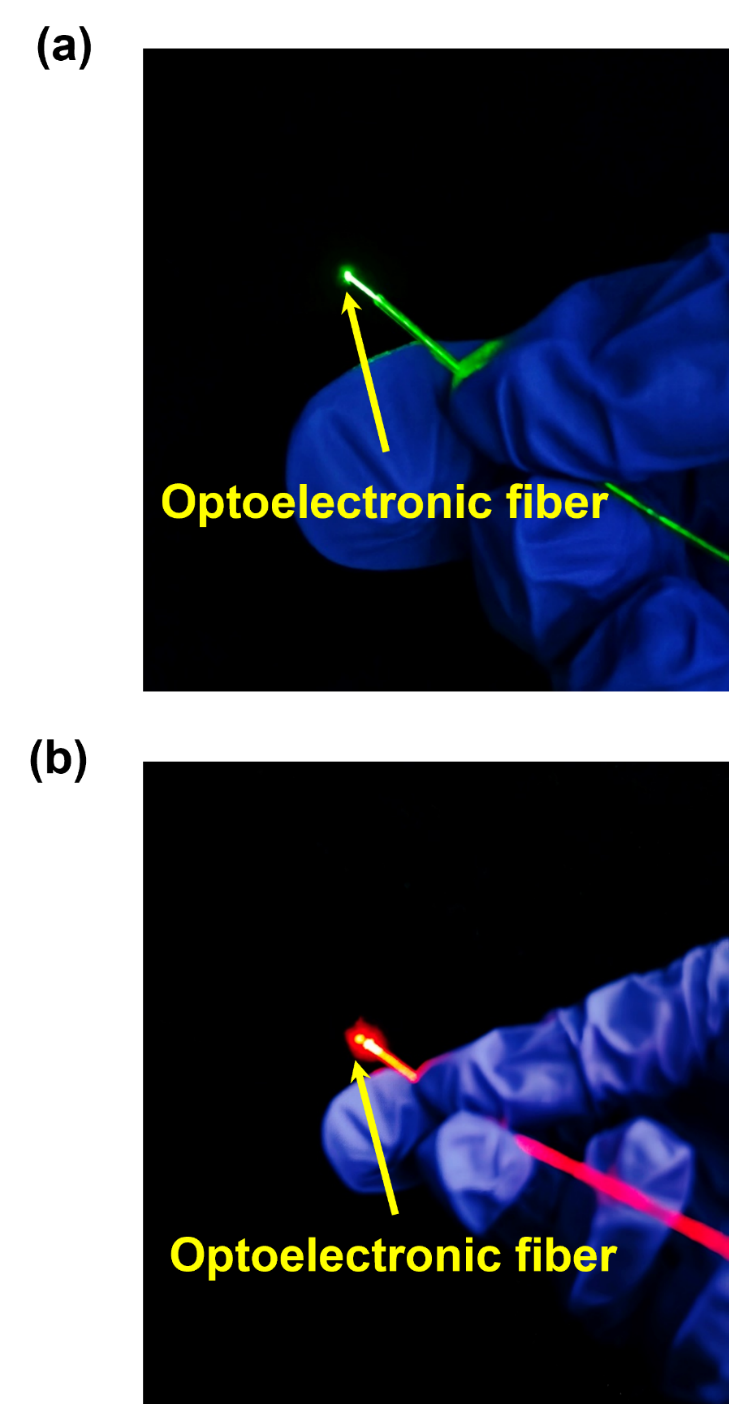


**Figure S5** The photos of optoelectronic fiber under 532 nm (a) and 635 nm (b) laser excitation, respectively. Under 532 nm and laser 635 nm excitation, the optoelectronic fiber demonstrates efficient light transmission capabilities, as the laser beam propagates clearly along the fiber length.

**
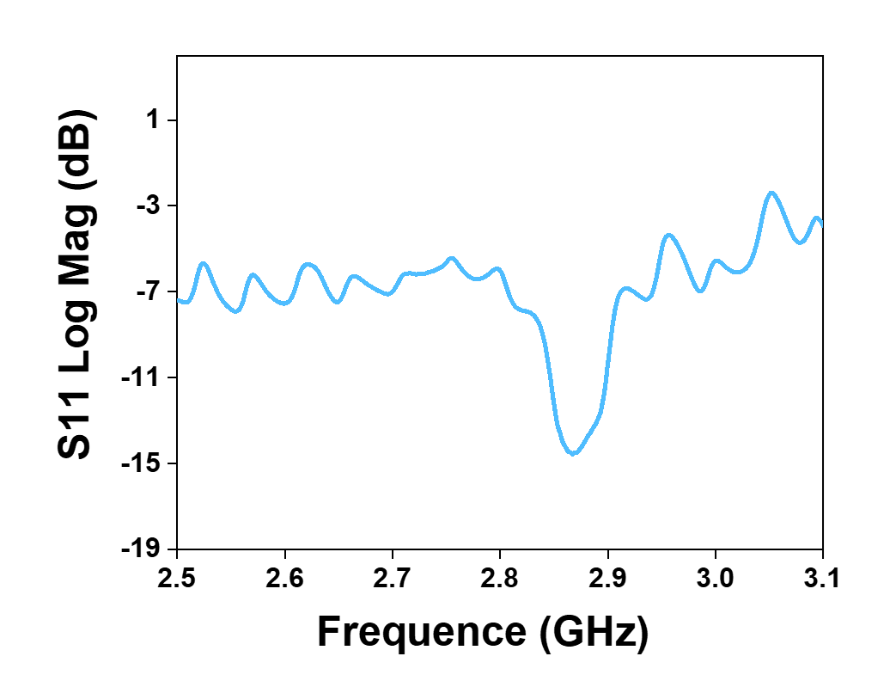
**

**Figure S6** Measured S11 parameter of the microwave transmission structure. Measured microwave reflection coefficient (S11) of the optoelectronic fiber device. The measured S11 parameter reaches -14.63 dB at ~2.87 GHz, indicating efficient microwave coupling and loading into the transmission structure.

**
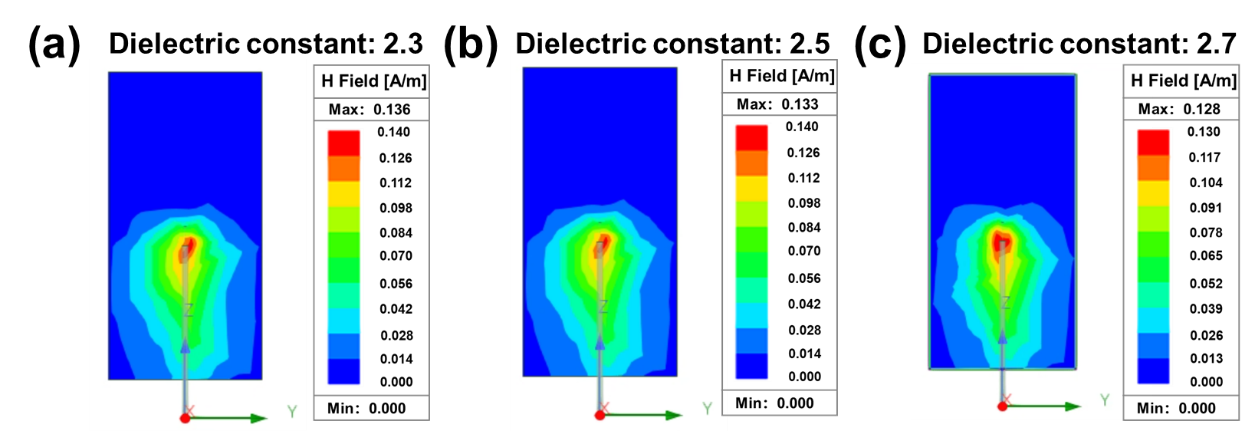
**

**Figure S7** HFSS simulation of microwave magnetic field distribution. Simulated microwave magnetic field distribution for different dielectric constants (a) $\varepsilon$ with 2.3, (b) $\varepsilon$ with 2.5 and (c) $\varepsilon$ with 2.7. The small variation indicates that the microwave field is robust against dielectric variations. In all cases, the magnetic field is concentrated near the transmission line and decays rapidly with distance.


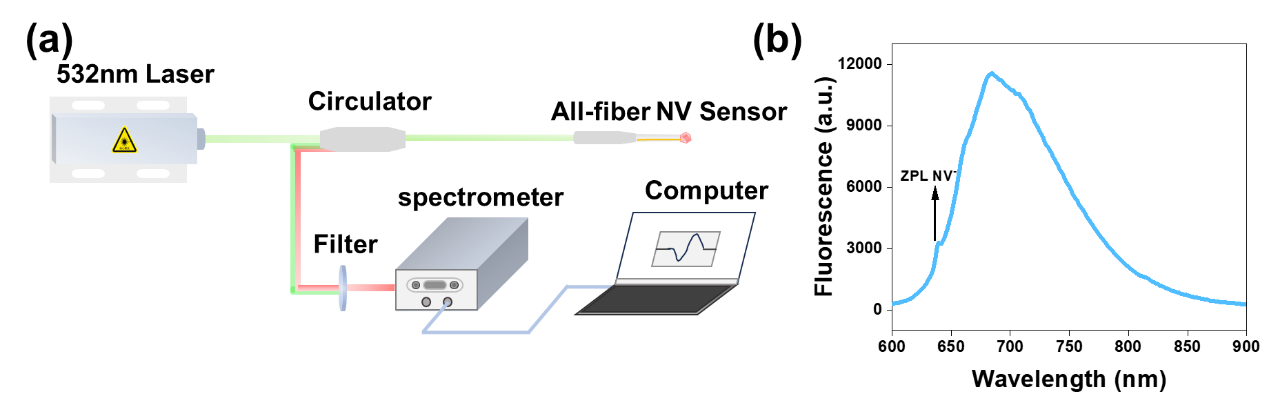


**Figure S8** (a) Fluorescence spectroscopy optical path diagram, (b) The fiber-based photoluminescence (PL) spectrum of the NV centers was measured through the complete fiber-optic probe system under 532 nm laser excitation. The excitation light was coupled into the fiber to stimulate the NV centers located at the fiber tip, and the emitted fluorescence was collected and guided back through the same fiber. The signal was then routed via a fiber circulator and passed through a 600 nm long-pass filter before being analyzed using a spectrometer (AVANTES AvaSpec-ULS2048XL-EVO). The measured spectrum exhibits a pronounced zero-phonon line (ZPL) at approximately 637 nm, corresponding to the radiative transition of negatively charged NV⁻ centers from the excited state to the ground state. This is accompanied by a broad phonon sideband extending to longer wavelengths (>650 nm), arising from lattice vibrations in the diamond. The clear observation of these spectral features confirms the successful integration and optical activation of NV⁻ centers within the diamond, as well as the capability of the fiber probe to efficiently excite and collect fluorescence for spectroscopic readout.

**
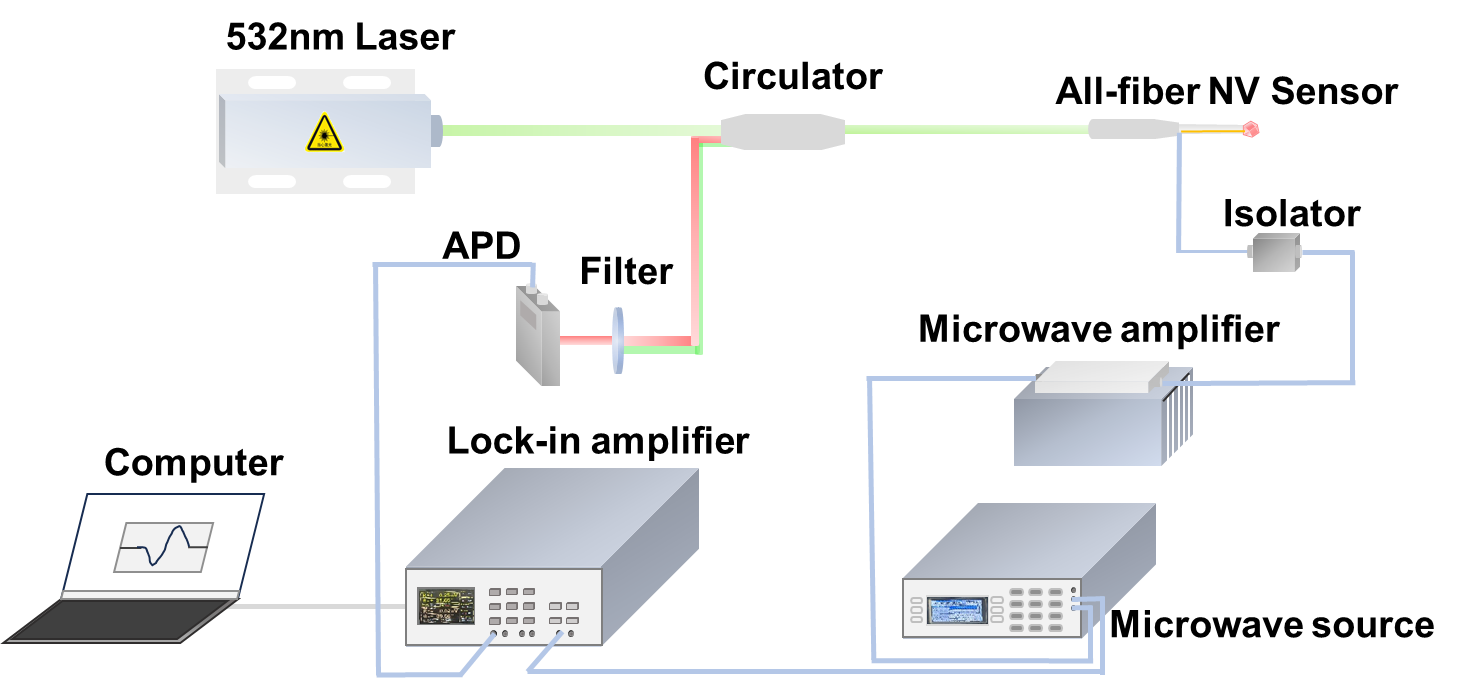
**

**Figure S9** Diagram of test system for the quantum fiber probe. The test system employed for the quantum fiber probe is shown in Figure S7. A 532 nm laser (MGL-FN-532, CNI Optoelectronics) couples into the quantum photonic thermal fiber system via a fiber circulator (WMC3L1S, Thorlabs). The fluorescence signal undergoes spectral purification using a long-pass filter (FELH0600, Thorlabs, >600 nm cutoff) before intensity measurement via an avalanche photodiode (APD410A/M, Thorlabs). Microwave delivery employs the fiber-embedded copper electrode as a radiative antenna, fed by a microwave source (SMB100A, Rohde & Schwarz) and amplified (ZHL-16W-43-S+, Mini-Circuits) to drive NV spin transitions. A 50 Ω-terminated isolator protects the amplifier from reflected power while maintaining impedance matching. To enhance signal-to-noise ratio and acquisition speed, we implement phase-sensitive detection through frequency-modulated microwave excitation synchronized with a lock-in amplifier (OE1022D, Sine Scientific Instruments). The APD output connects to the signal input channel, enabling real-time demodulation of ODMR spectral features.


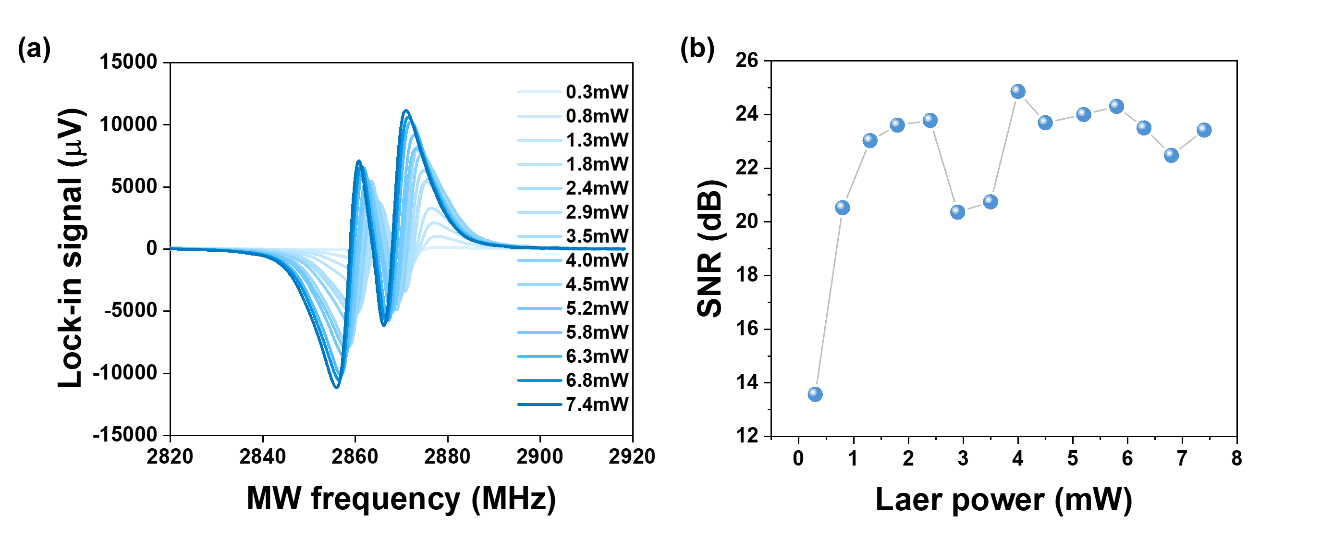


**Figure S1****0** Laser power dependence of ODMR performance. (a) ODMR spectra under different laser powers and (b) the dependence of Signal-to-Noise ratio on different laser power with microwave -2 dBm.


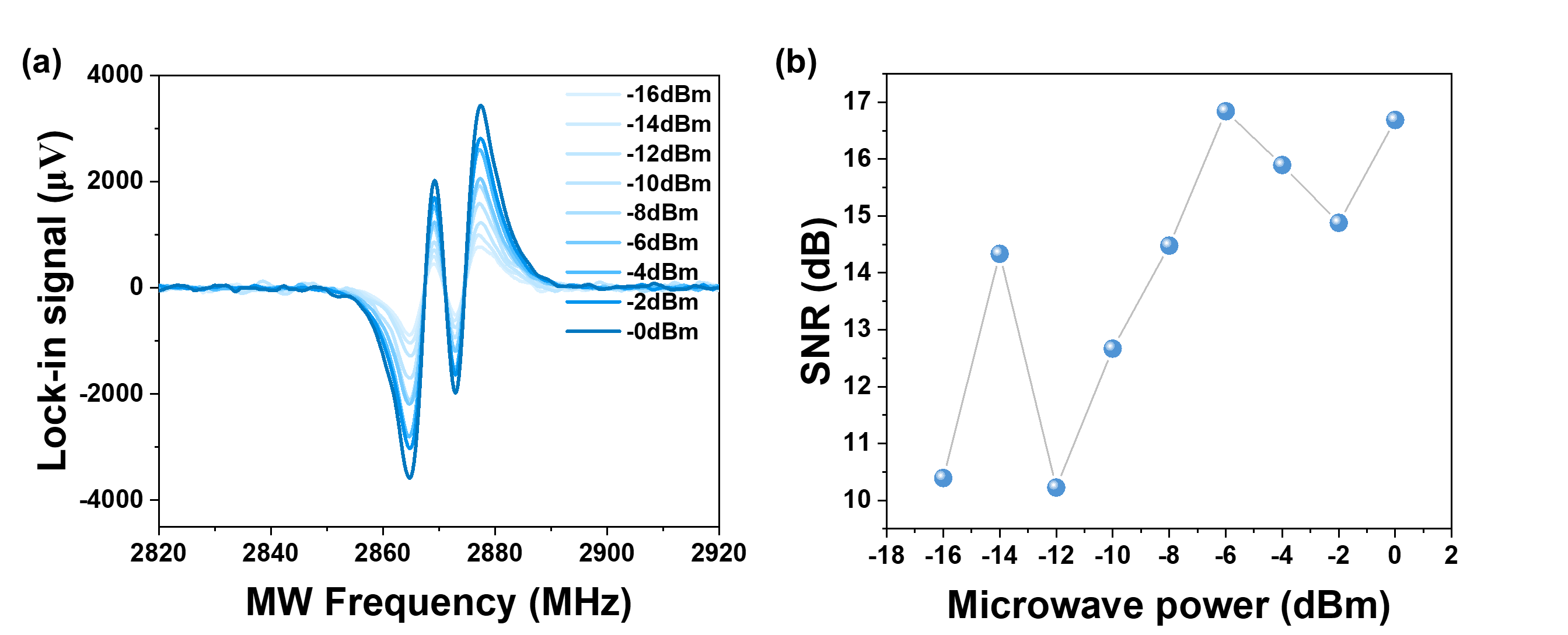


**Figure S11** Microwave power dependence of ODMR performance. (a) ODMR spectra under different laser powers and (b) the dependence of Signal-to-Noise ratio on different microwave power with laser power 0.8 mW.


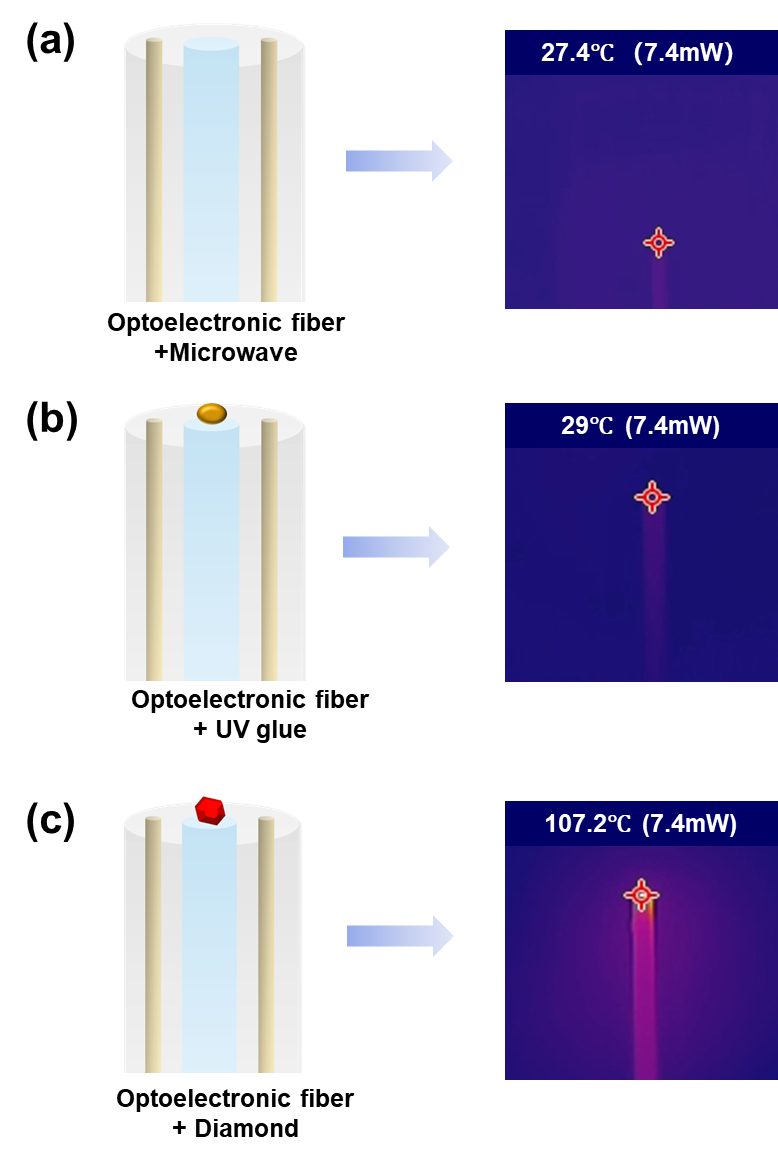


**Figure S12** Investigation of the thermal source of optoelectronic fiber quantum probes. To confirm that the temperature rise predominantly originates from the photothermal effect of the diamond NV centers, rather than heating from the microwave, optical fiber itself, or the UV-curable adhesive used during fabrication, we conducted a series of control experiments. (a) Optoelectronic fiber without diamond under microwave loading of 2dBm. (b) Optoelectronic fiber with UV glue. (c) Optoelectronic fiber with diamond. In these cases, 7.4mW green laser were illuminated to the end face through optoelectronic fiber. Through comparing the thermal distribution images, it is indicated that the photothermal effect predominantly arises from the diamond, rather than fiber itself, microwave or UV glue.

**
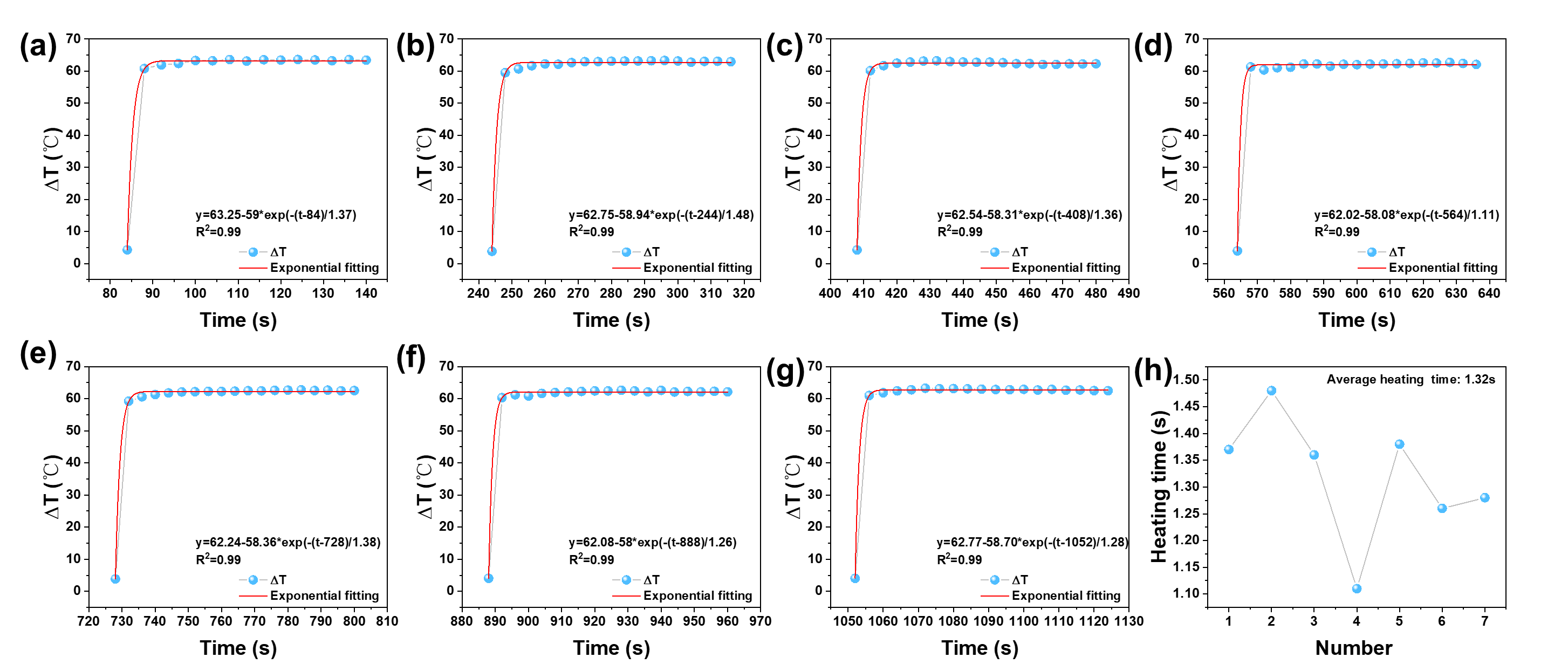
**

**Figure S13** (a-g) Experimental data on heating time for 7 cycles of the device and (h) extract data from the 7-cycle heating test.

**
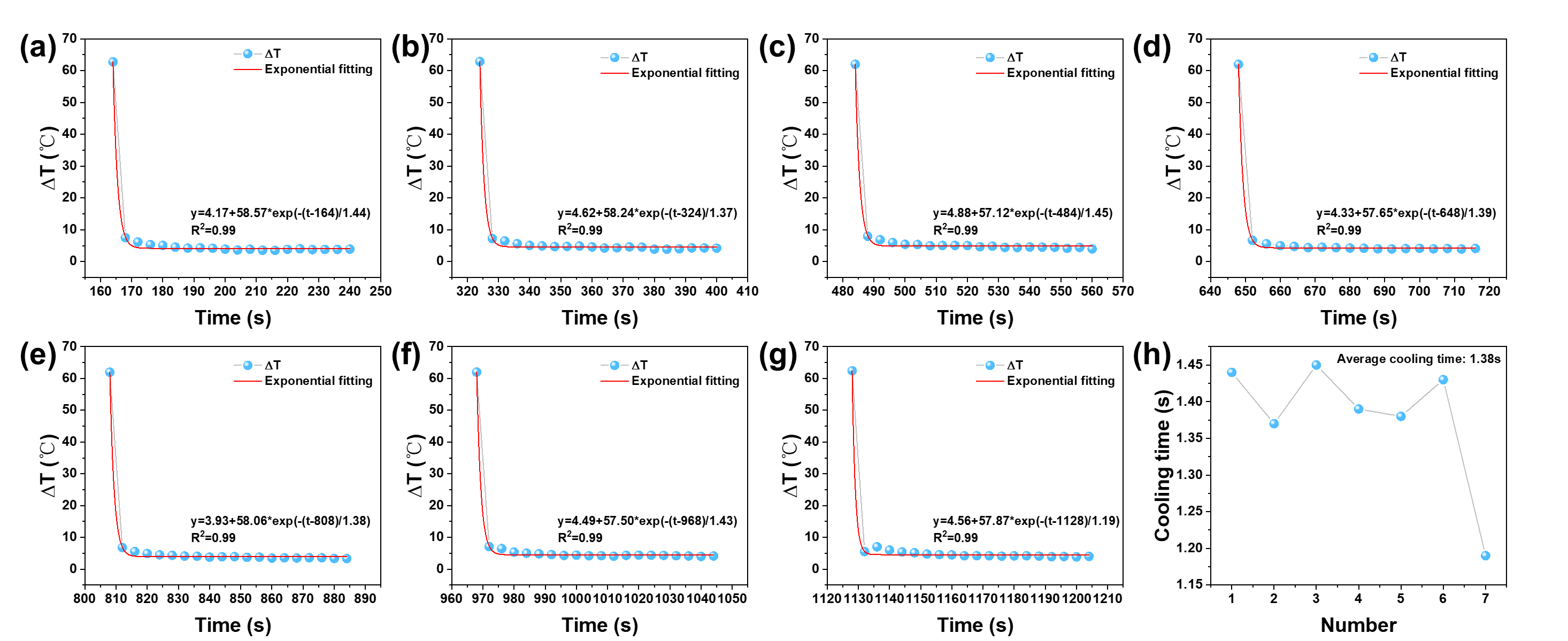
**

**Figure S14** (a-g) Experimental data on cooling time for 7 cycles of the device and (h) extract data from the 7-cycle cooling test.


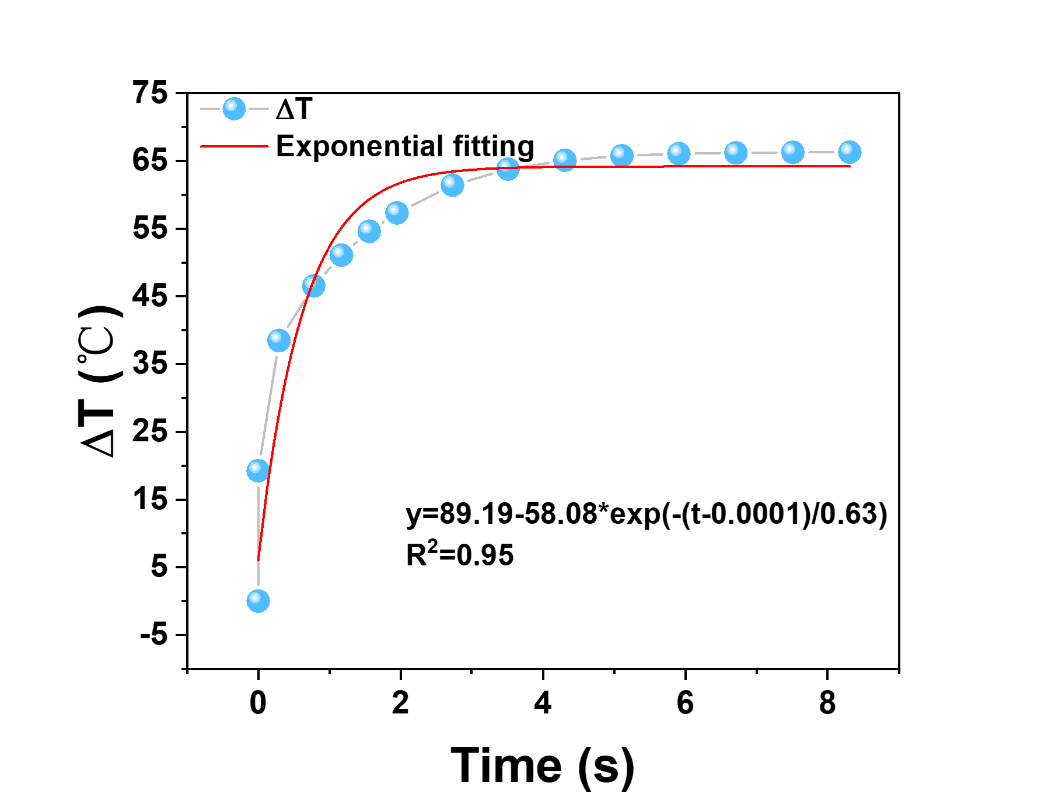


**Figure S15** Simulation data plots of transient thermal under 100 μm away from under air condition.

**
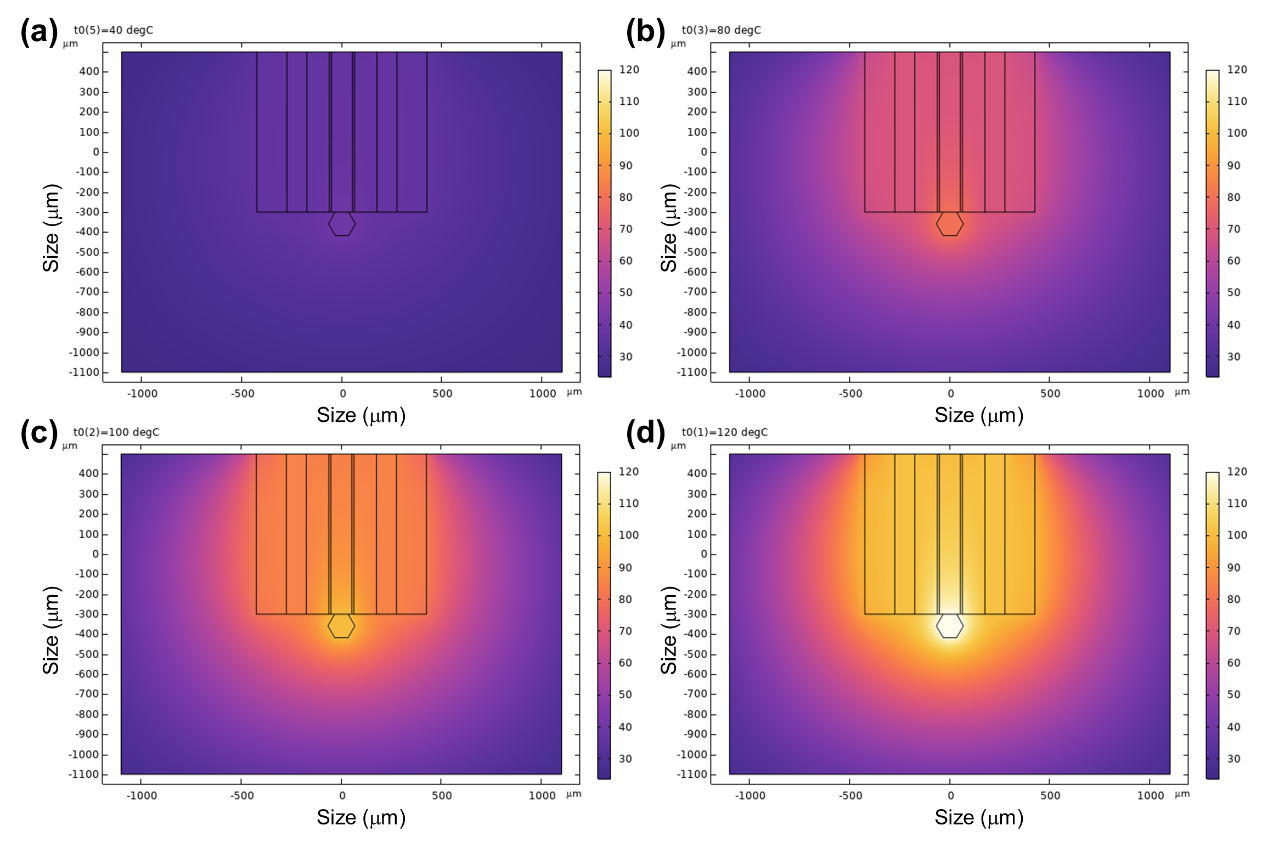
**

**Figure S16** Simulation data plots showing the thermal field distribution of diamond at (a) 40, (b) 80, (c) 100 and (d) 120°C.

**Figure S17** Biological application of quantum fiber probe for in vitro tissue photothermal ablation. (a) The infrared thermal images of the optoelectronic fiber composite diamond NV center under air (upper) and chicken tissue (down). (b) Histograms of air and chicken tissue generation temperatures at different laser powers. Infrared thermographic imaging of the fiber probe in air again showed laser power-dependent heating profiles and confirmed a high photothermal conversion efficiency of 11.1 °C/mW, which is much consistent with the self-monitored efficiency. After inserting the probe into the tissue, progressive increases in laser power induced localized temperature rises at the fiber–tissue interface reaching 25.6, 34.2, 47.7, and 69.1°. These results reveal a robust linear correlation between laser power and thermal output, yielding a tissue-specific conversion efficiency of 6.1°C/mW. The reduced slope compared to air-environment testing originates from enhanced thermal dissipation in biological tissue.

**
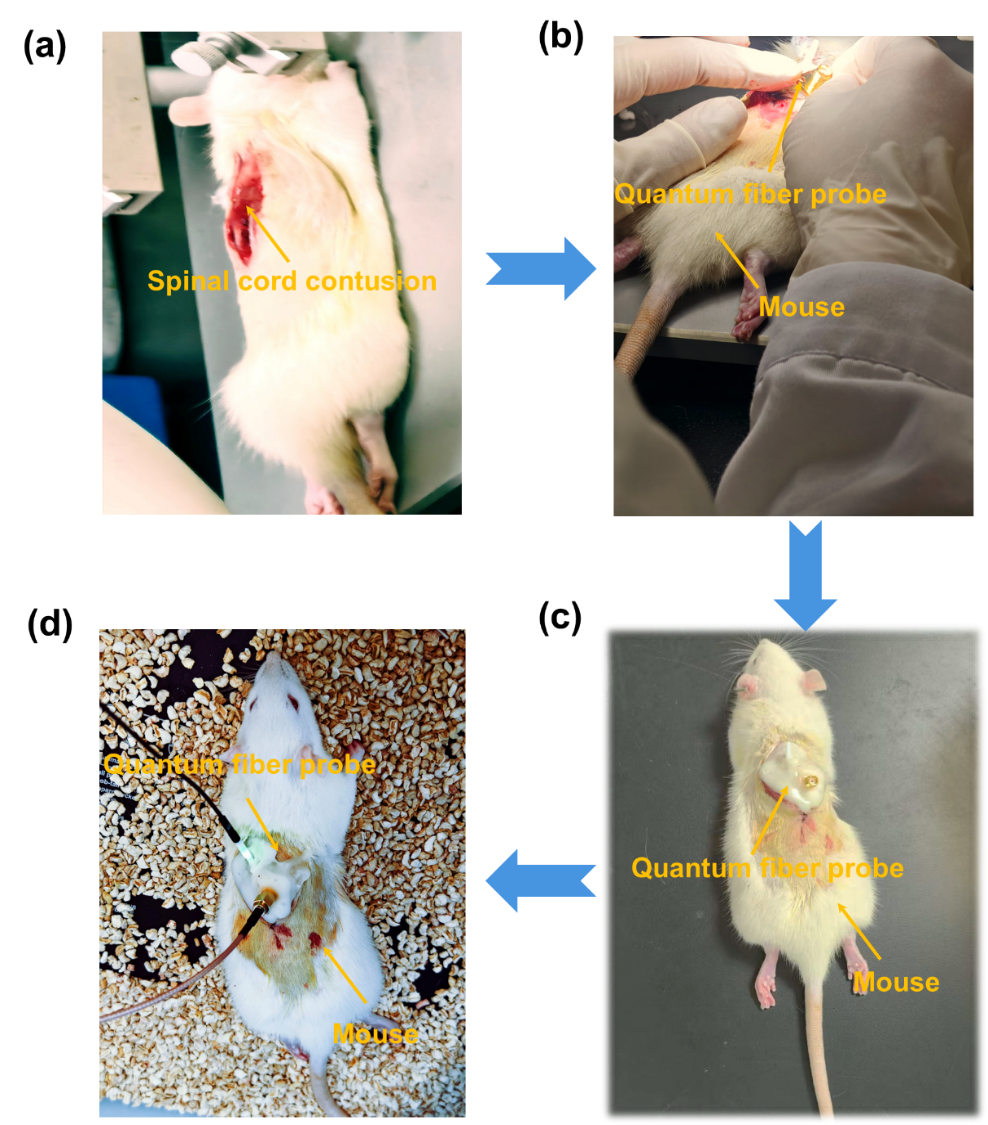
**

**Figure S18** The photos of the experimental process of using fiber quantum probes for in vivo spinal cord injury repair. (a) The mouse with spinal cord contusion. (b) Quantum fiber probe implantation. (c) The mouse after quantum fiber probe implantation. (d) Thermal therapy operation. The provided images depict a quantum thermal fiber probe successfully implanted within a live mouse, showcasing a critical step towards in-vivo biomedical applications. The ultra-thin, flexible fiber probe is shown minimally invasively inserted into target tissue, reduced physiological damage and stress to the animal. This setup is designed for precise, localized thermal therapy or hyperthermia treatment, leveraging the probe's ability to function as both a heater and a thermometer.

**
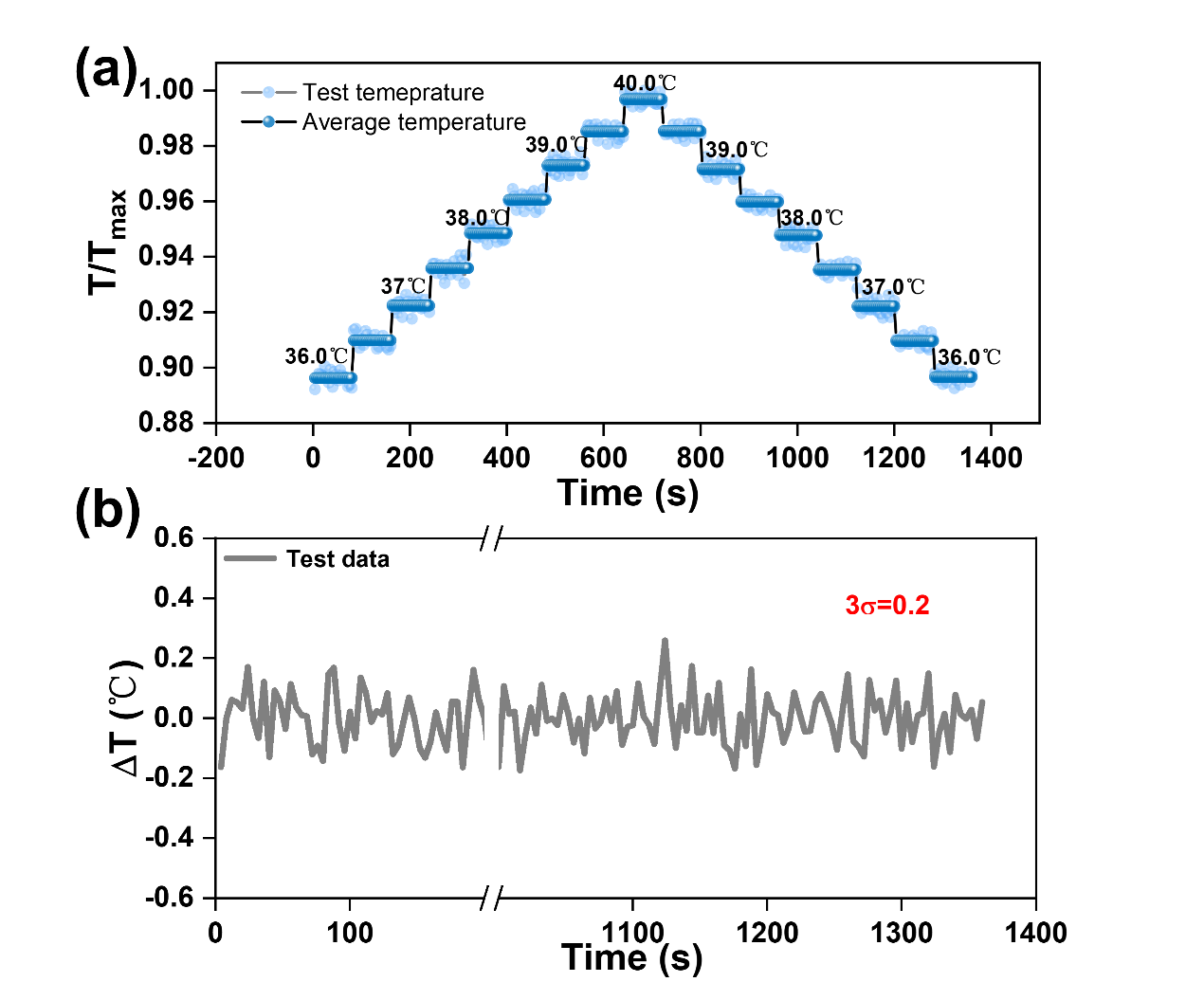
**

**Figure S19** The stability of the quantum thermal probe with small gradient temperature rang. (a) Ratio of measured temperatures to maximum. (b) The deviations of measured temperatures to the average values and the statistical results. The results indicate that precise temperature regulation within 0.5°C is achievable across the therapeutic range of 36-40°C, proving that the device has controllable accuracy in the biological heat treatment temperature range. Such precision is paramount in biological heat treatment applications, where even minor temperature fluctuations can significantly impact cellular viability and treatment efficacy. The data conclusively proves that the device operates with the necessary stability and controllability within the delicate thermal window required for hyperthermia therapy or other sensitive biomedical procedures, establishing it as a highly reliable tool for advanced *in vivo* thermal management.

**References:**

1. M. W. Doherty, N. B. Manson, P. Delaney, F. Jelezko, J. Wrachtrup, L. C. L. Hollenberg, Phys. Rep. **2013**, 528, 1.
2. A. Dréau, M. Lesik, L. Rondin, P. Spinicelli, O. Arcizet, J.-F. Roch, V. Jacques, Phys. Rev. B **2011**, 84, 195204.
3. J. M. Taylor, P. Cappellaro, L. Childress, L. Jiang, D. Budker, P. R. Hemmer, A. Yacoby, R. Walsworth, M. D. Lukin, Nat. Phys. **2008**, 4, 810.
4. J. F. Barry, J. M. Schloss, E. Bauch, M. J. Turner, C. A. Hart, L. M. Pham, R. L. Walsworth, Rev. Mod. Phys. **2020**, 92, 015004.
5. I. V. Fedotov, S. Blakley, E. E. Serebryannikov, N. A. Safronov, V. L. Velichansky, M. O. Scully, A. M. Zheltikov, Appl. Phys. Lett. **2014**, 105, 261106.
6. M. Fujiwara, H.-Q. Zhao, T. Noda, K. Ikeda, H. Sumiya, S. Takeuchi, Opt. Lett. **2015**, 40, 5702.
7. C.-C. Li, M. Gong, X.-D. Chen, S. Li, B.-W. Zhao, Y. Dong, G.-C. Guo, F.-W. Sun, Diamond Relat. Mater. 2017, 74, 119.V. M. Acosta, E. Bauch, M. P. Ledbetter, A. Waxman, L.-S. Bouchard, D. Budker, Phys. Rev. Lett. **2010**, 104, 070801.
8. M. W. Doherty, V. M. Acosta, A. Jarmola, M. S. J. Barson, N. B. Manson, D. Budker, L. C. L. Hollenberg, Phys. Rev. B **2014**, 90, 041201.
9. D. M. Toyli, D. J. Christle, A. Alkauskas, B. B. Buckley, C. G. Van de Walle, D. D. Awschalom, Phys. Rev. X **2012**, 2, 031001.
